# The evolution of surgical hip dislocation utilization and indications over the past two decades: a scoping review

**DOI:** 10.1007/s00264-023-05814-w

**Published:** 2023-04-27

**Authors:** Ahmed A. Khalifa, Tohamy G. Hassan, Mohamed A. Haridy

**Affiliations:** 1 Orthopaedic Department, Qena faculty of medicine and University Hospital, South Valley University, Kilo 6 Qena-Safaga highway, Qena, Egypt; 2 Faculty of Medicine for Girls, Al-Azhar University, Cairo, Egypt; 3 Ibri Regional Hospital, Ibri, Aldhahira Governorate Oman

**Keywords:** Surgical hip dislocation, Ganz hip dislocation, Scoping review

## Abstract

**Purpose:**

To assess the evolution of surgical hip dislocation (SHD) utilization over the past 20 years, concentrating mainly on the patients’ population (adults vs. paediatric), the hip conditions treated using this approach, and reporting on complications of this procedure.

**Methods:**

This scoping review was conducted according to Preferred Reporting Items for Systematic reviews and Meta-Analyses extension for Scoping Reviews (PRISMA-ScR) guidelines. A PubMed database search was performed using specific search terms for articles related to SHD published between January 2001 and November 2022.

**Results:**

Initial search revealed 321 articles, of which 160 published in 66 journals from 28 countries were eligible for final analysis. The number of publications increased by 10.2 folds comparing the period from 2001 to 2005 with 2018 to 2022. USA and Switzerland contributed to more than 50% of the publications. Case series studies represented the majority of publications (65.6%). Articles including adult patients represented 73.1% of the publications while 10% were on paediatric patients; however, there was 14 folds increase in publications on paediatric patients comparing the first with the last five years. Managing non-traumatic conditions was reported in 77.5% of the articles, while traumatic conditions in 21.9%. Femoroacetabular impingement (FAI) was the most treated non-traumatic condition reported in 53 (33.1%) articles. In contrast, femoral head fractures (FHF) were the most treated traumatic condition, which was reported in 13 articles.

**Conclusion:**

The publications on SHD and its usage for managing traumatic and non-traumatic hip conditions showed an increasing trend over the past two decades from worldwide countries. Its use in adult patients is well established, and its utilization in treating paediatric hip conditions is becoming more popular.

**Supplementary Information:**

The online version contains supplementary material available at 10.1007/s00264-023-05814-w.

## Introduction

Owing to the hip joint complex anatomy and various affecting conditions, many surgical approaches for managing various conditions have been proposed [1, 2]; however, choosing the proper approach depends on many variables, including the surgeon, the patient, the pathological condition, special instruments, and the working team familiarity. To call an approach as being “ideal,” it should be safe, simple, anatomic (follows internervous and inter-muscular planes), with limited soft tissue damage, then it should provide adequate exposure and visualization to the pathological area, which is the acetabulum and the femoral head in the case of the hip joint [1–4].

Derived by the thought of Crock in 1996 [5], who suggested that a method should be developed enabling atraumatic, blood supply preserving hip dislocation to treat early phase diseases, Ganz et al. introduced the concept of surgical hip dislocation (SHD) through a trochanteric flip osteotomy (TFO) which preserved the hip external rotators with further protection of the medial femoral circumflex artery (MFCA) which is considered the essential femoral head blood supply, in their initial series, they reported no cases of femoral head avascular necrosis (AVN) after a minimum two year follow-up in 213 patients [6].

The promising results by Ganz et al. [6], and the supporting anatomical studies proving SHD safety regarding hip joint vascularity [7, 8], led to the widespread adoption of SHD in treating various hip pathologies. The current scoping review aimed to assess the evolution of SHD utilization over the past 20 years, concentrating mainly on the patients’ population, the hip conditions treated using this approach, and reporting on complications of this procedure. We hypothesized that an increasing trend in the number of publications and the utilization of SHD in traumatic and non-traumatic conditions, both in paediatric and adult patients, occurred over the past two decades.

## Methods

This scoping review was conducted according to Preferred Reporting Items for Systematic reviews and Meta-Analyses extension for Scoping Reviews (PRISMA-ScR) guidelines [9]. A PubMed database search was performed for articles published between January 2001 and November 2022; the search was limited to the English language and studies on humans using the following search strategy: (((surgical hip dislocation[Title/Abstract]) OR (hip surgical dislocation[Title/Abstract])) OR (Ganz surgical hip dislocation[Title/Abstract])) OR (safe surgical dislocation[Title/Abstract]). We included articles reporting results from clinical situations (original studies [randomized controlled trials (RCTs), cohort, and case series], case reports, and technical notes) where the desired data were reported and could be extracted. We excluded review articles (systematic and narrative), studies lacking data of interest (cadaveric, radiological, and biomechanical studies), editorials, commentaries, and if the data of interest were not reported clearly. The search results were downloaded from PubMed in a detailed abstract and reference format. All article titles and abstracts were screened for eligibility. The following characteristics of the included studies were extracted into Microsoft Excel 2016 (Microsoft, Washington, United States): title, authors, year of publication, country of origin, condition treated (traumatic vs. non-traumatic), the patient population included (paediatric vs. adults) where patients aged <16 years old were considered paediatric based on the definition proposed by Michelson and Neuman [10], journal publishing the article, PubMed Identifier (PMID), and Digital Object Identifier (DOI).

## Results

### Search strategy

**Fig. 1:**
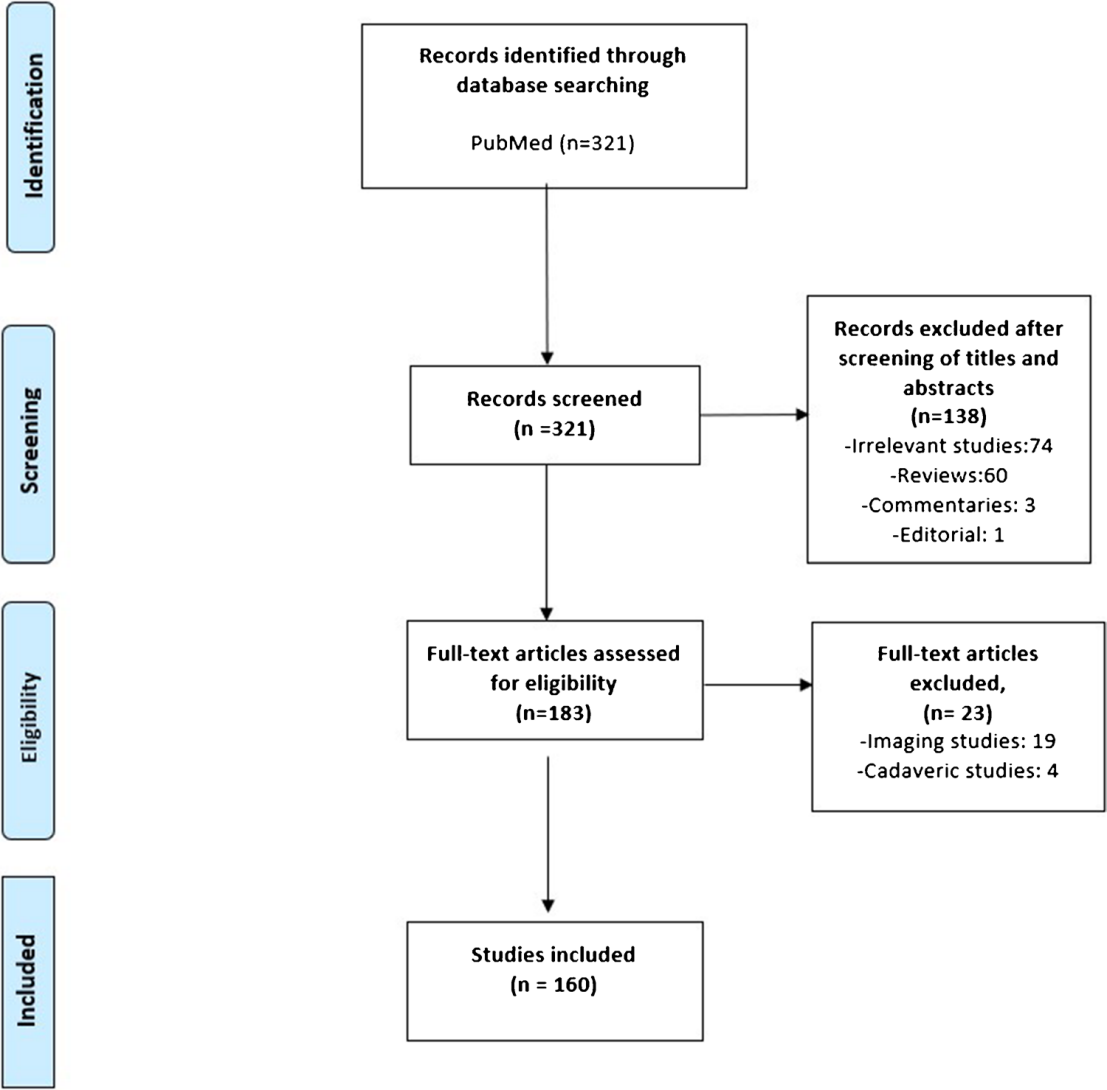
PRISMA chart of the search strategy

Initial search revealed 321 articles, of which 160 were eligible for final analysis. The PRISMA flow diagram illustrates the search results (Fig. 1). The details of the included articles are shown in Supplementary file 1.

### Study characteristics

**Fig. 2:**
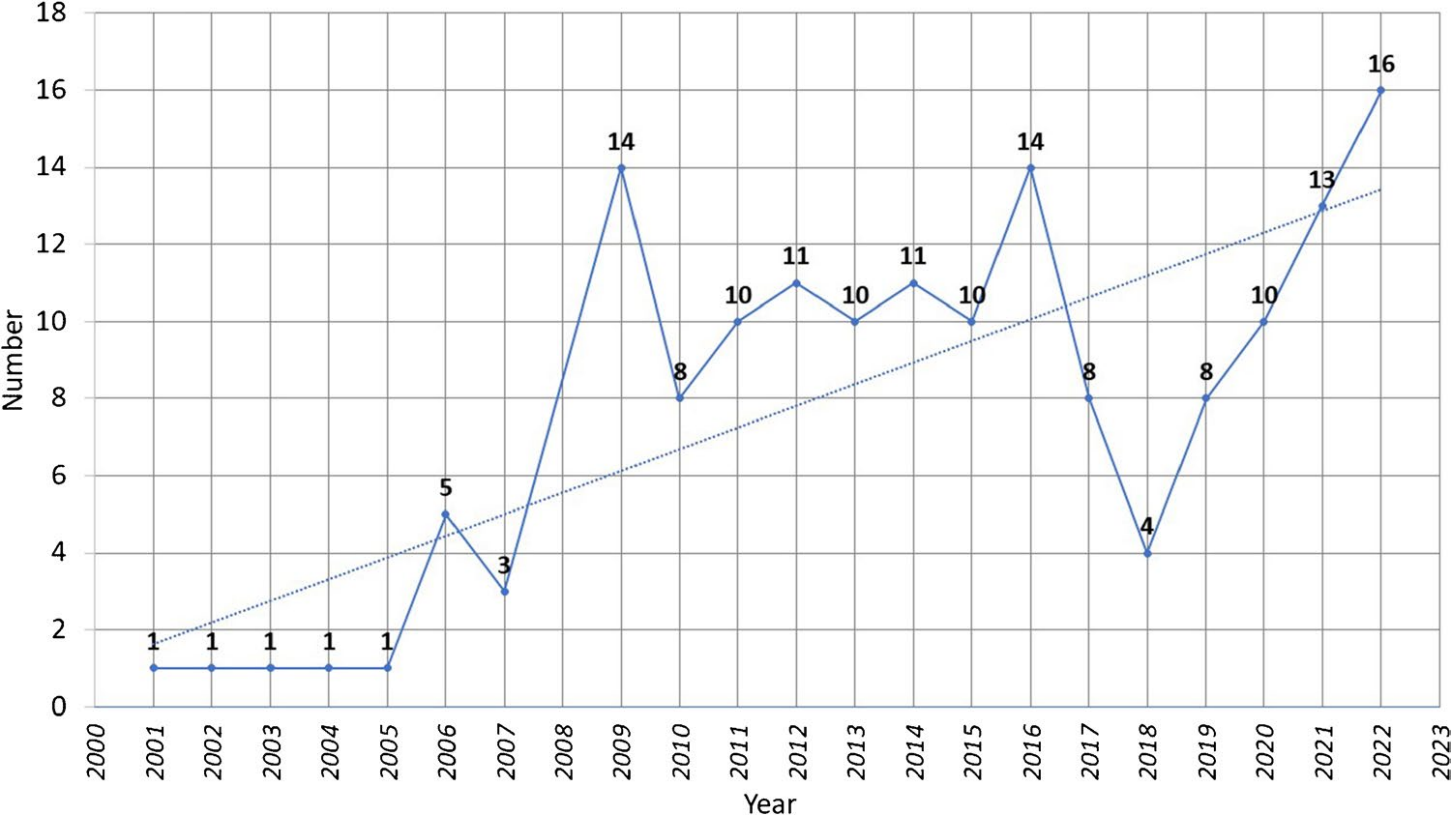
Number of articles published per year

The number of publications showed an increasing trend over time, where one study was published in 2001 compared to 16 articles published in 2022, furthermore comparing the number of articles published in the first five years (2001 to 2005) to articles published in the last five years (2018 to 2022) showed an increase by 10.2 folds (Fig. 2).

### Country of origin

**Fig. 3:**
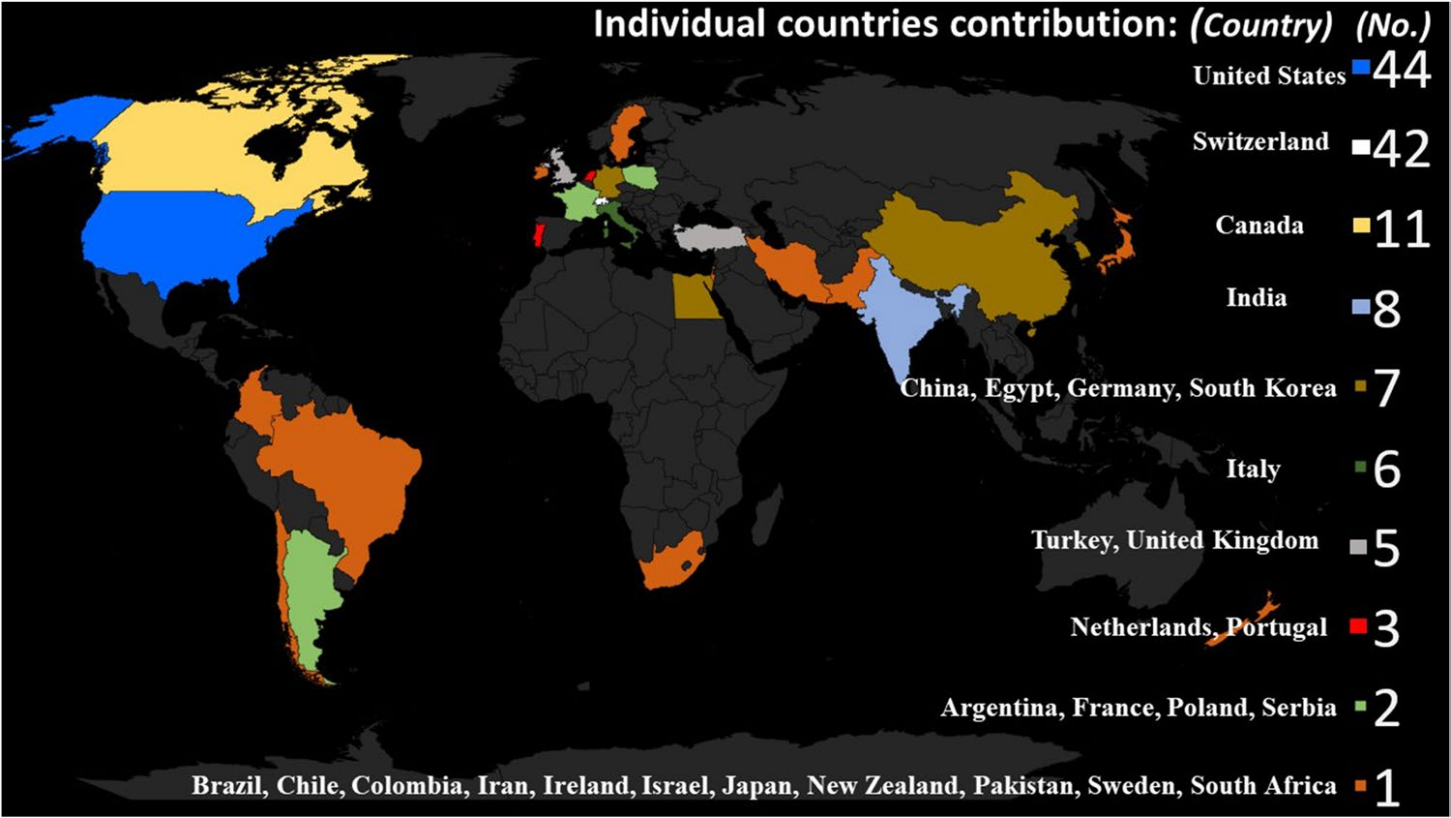
Individual countries’ contribution to the authorship of the published articles

The articles originated from 28 countries; in 13 articles, there was cooperation between authors from two countries. USA and Switzerland contributed over 50% of the publications (82 articles) (Fig. 3).

### Study type

**Fig. 4:**
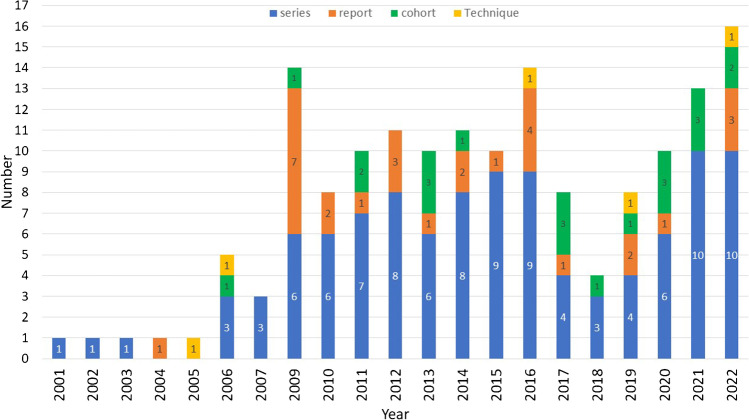
Various article types published per year

Regarding the type of studies included, case series represented the most common type, 105 articles (65.6%), followed by case reports 29 (18.1%), cohort studies (comparative studies) 21 (13.1%), and technical notes five (3.1%) (Fig. 4).

### Included patient population

**Fig. 5:**
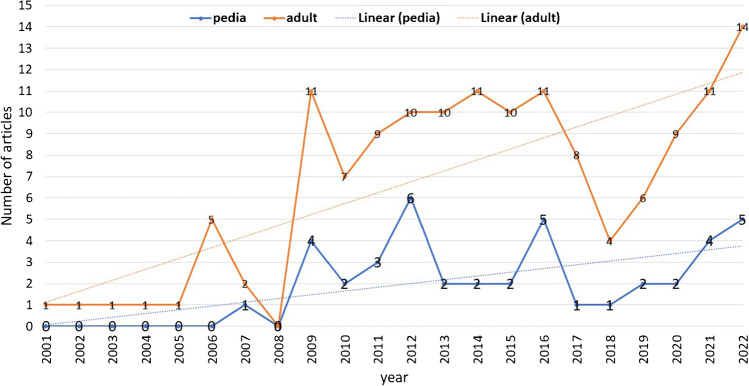
Number of articles published on adult or paediatric patients per year

In the first publication on SHD by Ganz et al. in 2001, the included population was above 16 years old (adults); however, over time, SHD was utilized in the paediatric population, which showed an increasing trend over the review period. The included studies were reported on adults only in 117 (73.1%), mixed in 26 (16.3%), and paediatric only in 16 (10%), and in one study, the authors did not report the age of the included patients. So, the SHD performed in adults was included in 143 (89.4%) and paediatric in 42 (26.5%) articles. There was an increase of 14 folds in the publications on the paediatric population comparing the first and the last five years (Fig. 5).

### Conditions treated

**Fig. 6:**
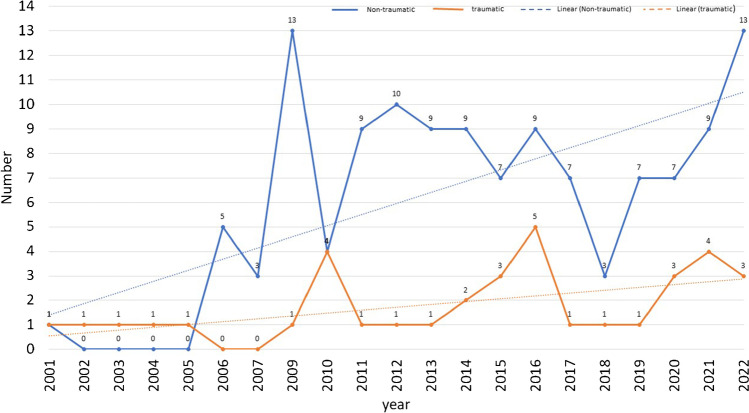
Number of articles published on traumatic and no-traumatic conditions per year

We classified the conditions treated in the included articles into either traumatic or non-traumatic conditions. In 124 (77.5%) articles, the conditions were only non-traumatic; in 35 (21.9%) articles, the conditions were only traumatic; and in one (0.6%) article, the authors reported treating both conditions. The number of articles treating different conditions over the review period is shown in (Fig. 6), and the various included conditions and their treatment strategy are mentioned in (Table 1). In both paediatric and adult populations, reports on the treatment of non-traumatic conditions far exceeded those of traumatic conditions, 38 and four in paediatrics and 111 and 33 in adults, respectively. Femoroacetabular impingement (FAI) was the most commonly treated non-traumatic condition reported in 53 (33.1%) articles. In contrast, femoral head fractures (FHF) were the most commonly treated traumatic condition reported in 13 (8.1%) articles.

**Table 1:** Details the various traumatic and non-traumatic conditions treated through SHD and the treatment strategy performed for each

*Non-traumatic condition*	*Management options*	*Traumatic conditions*	*Management options*
Femoro-acetabular Impingement (FAI)		*Acetabular fracture*	Open reduction and internal fixation (ORIF)
*FAI* with or without labral damage, including CAM or Pincer types- coxa profunda- the sequel of SCFE	Either one or a combination of the following procedure according to the pathology present: osteochondroplasty -labral repair or reconstruction- Osteochondral Autologous Transfer- acetabular rim trimming - periacetabular redirectional osteotomy (PAO)		
Femoral head chondral defect			
*Avascular necrosis (AVN) of the femoral head*	Partial resurfacing using Hemi-cap, impaction bone grafting, femoral neck rotational osteotomy, femoral neck varus osteotomy, fresh osteochondral allograft, AMIC	*Femoral head fractures (FHF) various Pipkin types*	ORIF
*Osteochondritis dissecans (OCD) of the femoral head*	Grafting- mosaicplasty- Osteochondral autologous transfer (OATS)	*FHF with Osteochondral Injury*	Osteochondral Autograft from the Ipsilateral Femoral Head- Autologous Osteochondral Transfer (Mosaicplasty)
		*Irreducible hip dislocation*	Open reduction and capsule-labral complex repair-management of intraarticular pathology accordingly
Excision of an intraarticular mass			
*Femoral head Chondroblastoma*	Excision and grafting or cement		
*Femoral head osteochondroma*	excision		
*Femoral head Giant Cell Tumor (GCT)*	Excision and bone cement or grafting		
*Acetabulum osteochondroma*	Excision		
*Acetabulum osteoblastoma*	curettage and bone cement		
*Acetabulum Osteoid osteoma*	Excision	*Femoral head transphyseal Fracture*	ORIF
*Ligamentum teres Fibromyxoid pseudotumor*	standard osteochondroplasty of the head-neck junction, fresh osteochondral allograft	*Femoral head impaction fracture*	Osteochondral Transplantation
*Femoral neck osteochondroma*	Excision		
		*Posttraumatic labral interposition*	Labral repair
Paediatric hip conditions and hip deformities			
*Neglected DDH*	femoral shortening and capsular arthroplasty- redirectional PAO		
*Slipped Capital Femoral Epiphysis (SCFE)*	Modified Dunn Procedure- corrective neck osteotomy- open reduction and internal fixation using an intracapsular osteotomy	*Recurrent traumatic hip dislocation*	Refixation of the peri-osteolabral complex, capsular closure, anterior femoral osteochondroplasty combined with debridement of the anterior chondral flap
*FAI after SCFE*	modified Dunn procedure	*Traumatic femoral head chondral defects*	Transfer of osteochondral shell autografts
*Caput Flexum Deformity of the Hip*	anterior open wedge femoral neck osteotomy-		
*Legg-Calvé-Perthes*	osteochondroplasty and relative neck lengthening- Morscher's femoral neck lengthening osteotomy- PAO and intertrochanteric osteotomy		
*Various conditions*			
*Pigmented villonodular synovitis (PVNS)*	synovectomy		
*Intraarticular Retained bullet*	Extraction		
*Synovial chondromatosis*	synovectomy and removal of loose bodies		
*Various hip pathologies (osteoarthritis (OA)- AVN)*	hip resurfacing		
*Malunited acetabular fracture*	Reduction osteotomy		

### Publishing journals

**Fig. 7:**
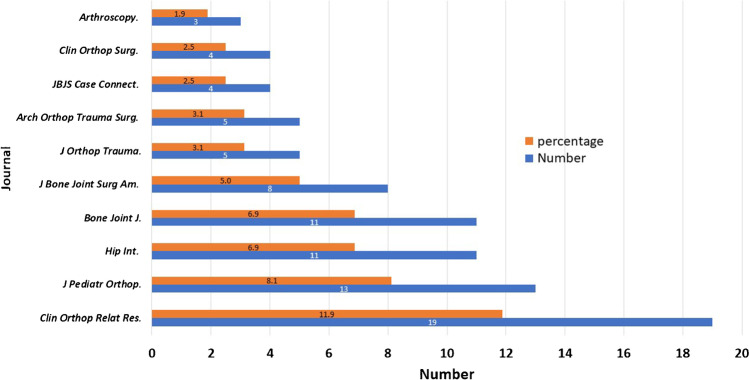
Top ten publishing journals

A total of 66 journals participated in publishing the included articles; the top ten journals published 51.9% (83) of the articles (Fig. 7).

## Discussion

The current review showed an increasing trend in publications discussing and reporting on SHD over the past 20 years from authors worldwide concerned with treating traumatic and non-traumatic conditions. Also, its use in adult patients is well established, and applying this approach to paediatric patients is becoming more popular and has increased tremendously over time.

Providing 360 degrees view of the acetabulum and the femoral head while preserving hip joint vascular supply made SHD approach a revolution in hip surgery. Furthermore, the efficacy and safety of utilizing this approach in managing traumatic and non-traumatic hip joint conditions were well documented in the literature [11–17].

Although Ganz et al. reported that they have been using SHD since 1992 [6]; however, after carrying out a PubMed search using the same search terms we used in the current review to check if there were any publications on SHD in the 10 years before 2001, the search revealed no results. This indicates that SHD was not generally accepted by the orthopaedic community until the initial results concerning its safety were published in 2001 [6], which encouraged wider adoption by more surgeons.

Furthermore, as the initial description was titled “Surgical dislocation of the Adult hip,” [6], we noticed that it was not until 2007 that the first article discussing the utilization of SHD in paediatric patients was published [18], followed by an increasing trend over the following years. Furthermore, intra-articular osteotomies to treat paediatric hip conditions were revolutionized after the addition of the extended retinacular flap to the SHD [19]. In a questionnaire study performed on members of the Paediatric Orthopaedic Society of North America by Thawrani et al. investigating their patterns of treating SCFE, the authors found that surgeons with less than 15 years of experience, those who work in academic practice, and surgeons treating a greater number of SCFEs are utilizing SHD more often to reduce the slip acutely [20].

Worth mentioning that the application of SHD in paediatric patients varied among studies, where some applied the same surgical technique as reported in adults [21, 22]; however, Smith et al. raised a concern regarding the possible injury of the proximal femoral physis and greater trochanter apophysis while performing TFO to achieve surgical hip dislocation, so the authors advocated performing SHD without TFO in patients younger than eight years old by elevating a small cartilaginous sleeve from the greater trochanter apophysis with the vastus lateralis, and abductor muscles still attached [23].

Several factors enabled SHD to be more popular among orthopaedic surgeons over time:

First, the lower incidence of AVN compared to other approaches when treating traumatic or non-traumatic hip conditions [6, 12], was owed to the preservation of hip external rotators, which subsequently protect the MFCA and performing a Z-shaped capsulotomy which allows raising a retinacular flaps, which contributes to blood supply preservation [6, 7, 24]. Furthermore, the vascularity status could be evaluated intraoperatively through a small perforation in the head, and a bleeding sign was correlated positively with the femoral head viability [6, 25]. Some authors described using intraoperative laser Doppler flowmetry as proposed by Nötzli et al. [26].

Second, the generous 360 degrees visualization of both the acetabulum and the femoral head, with further unobscured visualization of the hip central compartment, enables full assessment and treatment of labral and chondral injuries, as well as excision of various lesions and fixation of fractures [13, 25]. Moreover, as FAI was the most commonly treated condition through SHD, this approach enables easy visualization of osteochondroplasty. It was also efficient in addressing atypical and posterior rim and cam FAI, which are difficult to be treated arthroscopically [27].

Third, when dealing with sequelae of paediatric hip conditions such as Perthes disease and SCFE, which are often associated with relative femoral neck shortening, which could lead to the development of ischia-femoral or trochanteric pelvic FAI, distalization of the TFO could achieve relative neck lengthening with further improving the mechanical properties of the hip abductor complex [28].

Fourth, Ganz et al. reported treating various conditions, which were mainly non-traumatic conditions related to FAI (84%), sequelae of Perthes’ disease (11.3%), and pigmented villonodular synovitis, synovial chondromatosis or cartilaginous exostosis (4.7%) [6]; however, the current review proved that SHD showed great versatility for managing various traumatic and non-traumatic conditions over time which are difficult to treat arthroscopically or through other hip approaches [27].

Although SHD is an appealing approach with a wide margin of safety, however, it carries some complications;

First is the relatively high incidence of heterotopic ossification (HO) formation; Ganz et al. reported an HO incidence of 37%, and two patients diagnosed with HO grade III required surgical excision [6]. Kargin et al. evaluated 44 patients who underwent SHD for non-traumatic conditions; they reported an incidence of HO formation of 36.5% and lateral thigh pain of 28,8% [29].

Second, the unique complications associated with SHD are the TFO non-union and chronic lateral thigh pain from prominent screws fixing the TFO, with the possibility of further surgery either to fix a non-united TFO or to remove prominent lateral screws. Ganz et al. reported three (1.4%) cases with TFO non-union [6]. In a multicentre study by Sink et al., they evaluated 334 hips that underwent SHD with a minimum 12-month follow-up, although they reported no AVN; however, TFO non-union was reported in six hips (1.8%), all united after revision of the internal fixation [30]. Some authors reported an incidence of residual lateral thigh pain in up to 46% of the patients, which did not influence the clinical outcomes [29, 31].

To overcome the previously mentioned complications, some authors reported performing SHD without needing TFO. Shannon et al. [32] described performing SHD through a modified Hardinge approach in an adult patient for excising an osseocartilaginous lesion of the acetabulum and the femoral neck; without performing a TFO; instead, they exposed the hip capsule by elevation off 20% of the gluteus medius of the greater trochanter which was retracted anteriorly with the rectus femoris, and the hip was dislocated anteriorly by gentle hip external rotation. At one year follow-up, they reported good outcomes. The same previous approach (modified lateral) without TFO was used in a study by Schweitzer et al. [33], including six hips (five adults and one paediatric), and no complications or AVN were detected after a minimum of two years of follow-up. Furthermore, an anterolateral (Watson jones) approach to the hip without the need for TFO was described in a report by Louahem et al. [34] to treat 15- and 16-year-old patients presented with hip OCD.

Third, the issue of the learning curve and the need for special training before adopting SHD were not discussed thoroughly in the literature; however, in some reports, the authors mentioned their inability to utilize SHD owing to unfamiliarity [35]. Furthermore, Smith et al. stressed the point that SHD should not be done in paediatrics unless the surgeon is familiar with the SHD technique, hip joint anatomical variation, and has a good understanding of different pathologies [23].

The current review has some limitations; first, we only included one database in our search, which could deprive other publications not indexed in PubMed from being included. Second, additional bibliometric parameters, such as the number of citations for each article, were not included in the analysis. Third, we did not evaluate the quality of the included studies, such as their evidence level and the possible bias.

## Conclusion

Utilization and indications of surgical hip dislocation showed an increase over the past two decades, proving the successfulness of this approach for managing various hip joint-related pathologies. Publications on its usage showed an increasing trend from worldwide countries. Its use in adult patients is well established, and its utilization in treating paediatric hip conditions is becoming more popular. However, SHD still carries some unique complications and drawbacks, which could be attributed to complex hip joint anatomy and the demanding technicality of the approach; furthermore, as the exact learning curve a surgeon needs to perform SHD safely was not evaluated, we believe that it is not suitable for occasional or general orthopaedic surgeons unless they have enough training, becoming oriented with the anatomical variation, and has a good understanding of different pathologies.

### Supplementary information

ESM 1 (DOCX 97 kb)

## Data Availability

All the data related to the study are mentioned within the manuscript; however, the raw data are available with the corresponding author and will be provided up on a written request.
