# Supplementary material for: The evolution of surgical hip dislocation utilization and indications over the past two decades: a scoping review
Source: Int Orthop. 2023 Apr 27;47(12):3053–62. doi: 10.1007/s00264-023-05814-w (PMC10673723; doi:10.1007/s00264-023-05814-w)
Supplement: Supplementary file 1 — (DOCX 97 kb) [file 264_2023_5814_MOESM1_ESM.docx]

| **Details of the included studies for the synthesis of the current scoping review** | | | | | | | | | | | | | |
| --- | --- | --- | --- | --- | --- | --- | --- | --- | --- | --- | --- | --- | --- |
| **Number** | **Authors** | **number of authors** | **Year of publication** | **COUNTRY of origin** | **TITLE** | **study type** | **PATIENTS** | **Trauma v. Non-trauma** | **condition** | **Management** | **journal** | **DOI** | **PMID** |
| 1 | Zhou et al | 10 | 2022 | China | Clinical Outcome of Surgical Hip Dislocation Combined with Impacting Bone Grafts and Implanting Iliac Bone Flaps in the Treatment of Osteonecrosis of the Femoral Head: A Mid-term Retrospective Study | case series | adult | non-trauma | AVN | Impacting Bone Grafts | Orthop Surg | 10.1111/os.13295 | 35478435 |
| 2 | Yoon et al. | 5 | 2022 | Korea | Safety of surgical hip dislocation in femoral head fracture and dislocation (FHFD) and avascular necrosis risk factor analysis of FHFD: midterm results confirmed by SPECT/CT and MRI | case series | adult | trauma | FHF | ORIF | J Orthop Surg Res | 10.1186/s13018-022-03160-y | 35578301 |
| 3 | Wang et al. | 4 | 2022 | China | Surgical treatment of femoroacetabular impingement in a patient with Stickler syndrome: a case report | case report | adult | non-trauma | FAI | osteochondroplasty | Ann Transl Med. | 10.21037/atm-21-4784 | 35571386 |
| 4 | Tripathy et al. | 4 | 2022 | India | Safe surgical hip dislocation for acetabular osteoid osteoma excision | case report | Pediatric | non-trauma | osteoid osteoma | excision | BMJ Case Rep. | doi: 10.1136/bcr-2021-246025 | 35228220 |
| 5 | Leibold et al. | 5 | 2022 | Switzerland | Surgical hip dislocation with relative femoral neck lengthening and retinacular soft-tissue flap for sequela of Legg-Calve-Perthes disease | case series | adult | non-trauma | sequela of Legg-Calve-Perthes disease | relative femoral neck lengthening and retinacular soft-tissue flap | Oper Orthop Traumatol. | 10.1007/s00064-022-00780-9 | 35930024 |
| 6 | Laboudie et al. | 4 | 2022 | Canada | Does labral treatment technique influence the outcome of FAI surgery? A matched-pair study of labral reconstruction versus repair and debridement with a follow-up of 10 years | cohort | adult | non-trauma | FAI | According to the present pathology | J Hip Preserv Surg | 10.1093/jhps/hnac017 | 35854806 |
| 7 | Klosterman et al. | 5 | 2022 | USA | Treatment of Coxa Profunda With Open Surgical Hip Dislocation Rim Resection Cam Resection and Labral Reconstruction | Technique | adult | non-trauma | coxa profunda | Rim Resection Cam Resection and Labral Reconstruction | Arthrosc Tech. | 10.1016/j.eats.2022.03.042 | 36061463 |
| 8 | Hosny et al. | 3 | 2022 | Egypt | Management of femoral head fracture by Ganz surgical dislocation of the hip | case series | adult | trauma | FHF | ORIF | J Orthop Traumatol. | 10.1186/s10195-022-00643-w | 35538323 |
| 9 | Feroe et al. | 5 | 2022 | USA | Surgical Hip Dislocation for a Diagnostic Dilemma: Differentiating Synovial Chondromatosis and Pigmented Villonodular Synovitis | case report | adult | non-trauma | PVNS | Synovectomy | Iowa Orthop J. |  | 35821952 |
| 10 | Díaz-Dilernia et al. | 4 | 2022 | Canada and Argentina | Short-Term Results of Osteochondral Autologous Transfer and Femoral Neck Osteochondroplasty for the Treatment of Osteochondral Lesions of the Femoral Head and Concomitant Femoroacetabular Impingement Syndrome: A Case Series | case series | adult | non-trauma | FAI | Osteochondral Autologous Transfer and Femoral Neck Osteochondroplasty | Hip Pelvis | 10.5371/hp.2022.34.3.177 | 36299472 |
| 11 | Chen et al. | 7 | 2022 | China | Outcomes of surgical hip dislocation combined with bone graft for adolescents and younger adults with osteonecrosis of the femoral head: a case series and literature review | case series | adult | non-trauma | AVN | bone graft | BMC Musculoskelet Disord | 10.1186/s12891-022-05456-w | 35619082 |
| 12 | Chen et al. | 4 | 2022 | USA | Acetabular labral reconstruction with medial meniscal allograft: preliminary results of a new surgical technique | case series | adult | non-trauma | labral reconstruction with medial meniscal allograft after FAI | fresh-frozen medial meniscal allograft for labral reconstruction during surgical hip dislocation for correction of pincer FAI due to acetabular over coverage | Eur J Orthop Surg Traumatol. | doi: 10.1007/s00590-021-02986-2. | 34028622 |
| 13 | Baraka et al. | 6 | 2022 | Egypt | Morscher's femoral neck lengthening osteotomy through surgical hip dislocation approach for preservation of Perthes and Perthes-like deformities | case series | mixed | non-trauma | Legg-Calvé-Perthes disease | Morscher's femoral neck lengthening osteotomy | J Child Orthop | 10.1177/18632521221080477 | 35615389 |
| 14 | Ahern et al. | 6 | 2022 | Ireland | Clinical and radiological outcomes following surgical hip dislocation for pediatric hip pathologies a prospective cohort study | cohort | Pediatric | non-trauma | various pathologies | according to each condition | Surgeon | 10.1016/j.surge.2022.09.005 | 36307306 |
| 15 | Abo-Elsoud et al. | 4 | 2022 | Egypt | Surgical hip dislocation for treatment of femoral head chondroblastoma: efficacy and safety | case series | adult | non-trauma | Chondroblastoma | Excision and bone cement | Int Orthop. | doi: 10.1007/s00264-021-05264-2 | 34799777 |
| 16 | Abdelnasser et al. | 3 | 2022 | Egypt | Surgical hip dislocation in fixation of acetabular fractures: Extended indications and outcome | case series | adult | trauma | Acetabular fracture | ORIF | Injury | doi: 10.1016/j.injury.2021.09.054. | 34620471 |
| 17 | Youngman et al. | 9 | 2021 | USA | The Association of alpha Angle on Disease Severity in Adolescent Femoroacetabular Impingement | case series | mixed | non-trauma | FAI | according to the pathology | J Pediatr Orthop. | 10.1097/BPO.0000000000001703 | 33208669 |
| 18 | Trikha et al. | 4 | 2021 | India | An unusual pattern of posterior wall fracture of acetabulum | case series | adult | trauma | Acetabular fracture | ORIF | Eur J Orthop Surg Traumatol. | doi: 10.1007/s00590-020-02814-z. | 33099679 |
| 19 | Steppacher et al. | 5 | 2021 | Switzerland | Acetabular Cartilage Thickness Differs Among Cam Pincer or Mixed-Type Femoroacetabular Impingement: A Descriptive Study Using In Vivo Ultrasonic Measurements During Surgical Hip Dislocation | case series | adult | non-trauma | FAI | according to the pathology | Cartilage. | doi: 10.1177/1947603521990879. | 33550853 |
| 20 | Sarassa et al. | 5 | 2021 | Colombia | Long-Term Results of Slipped Capital Femoral Epiphysis Treated with the Modified Dunn Procedure in a Colombian Cohort | case series | Pediatric | non-trauma | SCFE | Modified Dunn Procedure | Clin Orthop Surg. | 10.4055/cios19038 | 34484635 |
| 21 | Risto et al. | 4 | 2021 | Sweden and UK | Outcome after osteochondroplasty and relative neck lengthening for patients with healed Legg-Calv-Perthes disease: a retrospective cohort study of patients with hip-deformities treated with osteochondroplasty and relative neck lengthening | cohort | adult | non-trauma | Legg-Calvé-Perthes | osteochondroplasty and relative neck lengthening with or without periacetabular osteotomy (PAO) | Hip Int. | 10.1177/1120700019896767 | 31875728 |
| 22 | Rego et al. | 6 | 2021 | Portugal and Switzerland | Femoral neck osteotomy in skeletally mature patients: surgical technique and midterm results | case series | adult | non-trauma | hip deformities | femoral neck osteotomy | Int Orthop. | 10.1007/s00264-020-04822-4 | 32997157 |
| 23 | Mundluru et al. | 8 | 2021 | USA | Acetabular Coverage May Affect Radiographic and Clinical Outcomes of Osteochondral Allograft Transplantation of Focal Femoral Head Lesions: A Case Series From a Single Institution | case series | mixed | non-trauma | Femoral head Osteochondral lesions | Osteochondral Allograft Transplantation | J Am Acad Orthop Surg Glob Res Rev. | 10.5435/JAAOSGlobal-D-20-00219 | 34637410 |
| 24 | Lieberman et al. | 6 | 2021 | USA | Patients With Symptomatic Sequelae of Slipped Capital Femoral Epiphysis Have Advanced Cartilage Wear at the Time of Surgical Intervention | cohort | adult | non-trauma | FAI | According to the pathology present | J Pediatr Orthop. | 10.1097/BPO.0000000000001797 | 33734202 |
| 25 | Khalifa et al. | 4 | 2021 | Egypt | Management of femoral head fractures through surgical hip dislocation (SHD): a demanding but safe technique | case series | adult | trauma | FHF | femoral head fracture treated through SHD | Arch Orthop Trauma Surg. | doi: 10.1007/s00402-020-03666-2. | 33113013 |
| 26 | Fang et al. | 8 | 2021 | China | Surgical hip dislocation for treatment of synovial chondromatosis of the hip | case series | adult | non-trauma | synovial chondromatosis | synovectomy and removal of loose bodies | Int Orthop. | doi: 10.1007/s00264-021-05045-x. | 33877408 |
| 27 | De Mauro et al. | 9 | 2021 | Italy | Gibson approach and surgical hip dislocation according to Ganz in the treatment of femoral head fractures | case series | adult | trauma | FHF | ORIF | BMC Musculoskelet Disord. | doi: 10.1186/s12891-021-04800-w. | 34789202 |
| 28 | Colares et al. | 7 | 2021 | Brazil | Intraoperative blood salvage in proximal femur epiphysiolysis surgical treatment with hip controlled dislocation technique: a case series study | case series | Pediatric | non-trauma | SCFE | Open reduction | Braz J Anesthesiol. | 10.1016/j.bjane.2021.02.061 | 34097945 |
| 29 | Aprato et al. | 4 | 2021 | Italy | Surgical hip dislocation vs arthroscopy for fixation of sub foveal femoral head fractures: A new technique for Pipkin type 1 fractures | cohort | adult | trauma | FHF | ORIF | Acta Biomed. | doi: 10.23750/abm.v92iS3.11724. | 34313665 |
| 30 | Xia et al. | 6 | 2020 | China | Comparison of surgical dislocation and impacting bone graft and surgical dislocation and rotational osteotomy for the treatment of ARCO III femoral head necrosis | cohort | adult | non-trauma | AVN | impaction bone graft and rotational osteotomy | Medicine (Baltimore). | 10.1097/MD.0000000000020215 | 32443350 |
| 31 | Steppacher et al. | 5 | 2020 | Switzerland | Surgical hip dislocation with femoral osteotomy and bone grafting prevents head collapse in hips with advanced necrosis | case series | adult | non-trauma | AVN | curettage, drilling and autologous bone grafting, Cartilage suturing, autologous matrix-induced chondrogenesis, an osteochondral autograft, Femoral osteotomy | Hip Int. | 10.1177/1120700019856010 | 31204506 |
| 32 | Shahien et al. | 4 | 2020 | USA | Surgical Hip Dislocation for Small Posterior Wall Fracture After Hip Subluxation | case report | adult | trauma | Acetabular fracture | ORIF | J Orthop Trauma. | 10.1097/BOT.0000000000001833 | 32639343 |
| 33 | Nepple et al. | 8 | 2020 | USA and Canada | Surgical Treatment of Femoroacetabular Impingement: Hip Arthroscopy Versus Surgical Hip Dislocation: A Propensity-Matched Analysis | cohort | adult | non-trauma | FAI | According to the pathology | J Bone Joint Surg Am | 10.2106/JBJS.20.00265 | 32925230 |
| 34 | Leibold et al. | 5 | 2020 | Switzerland | Femoral osteotomies for the treatment of avascular necrosis of the femoral head | case series | adult | non-trauma | AVN | femoral varus osteotomy | Oper Orthop Traumatol. | 10.1007/s00064-019-00642-x | 31784775 |
| 35 | Lamplot et al. | 5 | 2020 | USA | Open Reduction and Internal Fixation for the Treatment of Symptomatic Osteochondritis Dissecans of the Femoral Head in Patients With Sequelae of Legg-Calvé-Perthes Disease | case series | Pediatric | non-trauma | Osteochondritis Dissecans Sequelae of Legg-Calvé-Perthes Disease | ORIF of the femoral head osteochondral fragment | J Pediatr Orthop. | 10.1097/BPO.0000000000001192 | 32028473 |
| 36 | Hanke et al. | 5 | 2020 | Switzerland | Transfer of osteochondral shell autografts to salvage femoral head impaction injuries in hip trauma patients | case series | adult | trauma | traumatic femoral head chondral defects | Transfer of osteochondral shell autografts | Injury. | 10.1016/j.injury.2020.01.037 | 32033805 |
| 37 | Hancioglu et al. | 4 | 2020 | Turkey | Clinical and radiological outcomes of chronic severe slipped capital femoral epiphysis patients treated by surgical dislocation and modified Dunn osteotomy: Case series | case series | mixed | non-trauma | SCFE | modified Dunn osteotomy | Jt Dis Relat Surg. | 10.5606/ehc.2020.75101 | 32962595 |
| 38 | Gavaskar et al. | 6 | 2020 | India | Surgical Dislocation or the Modified Hueter Anterior Approach for Pipkin I and II Femoral Head Fracture Dislocations | cohort | adult | trauma | FHF | ORIF | J Orthop Trauma. | 10.1097/BOT.0000000000001877 | 32639395 |
| 39 | Faure et al. | 7 | 2020 | USA and Canada | Morscher Osteotomy Through Surgical Dislocation Approach for True Femoral Neck Lengthening with Greater Trochanter Transposition | case series | mixed | non-trauma | DDH and LCPD | Morscher osteotomies | J Bone Joint Surg Am. | 10.2106/JBJS.20.00405 | 32890044 |
| 40 | Shannon et al. | 4 | 2019 | USA | Surgical Hip Dislocation Using the Modified Hardinge Approach for Excision of Osseocartilaginous Lesions of the Acetabulum and Femoral Neck in an Adult: A Case Report | case report | adult | non-trauma | osteo-cartilaginous lesion acetabulum | Excision of Osteo-cartilaginous Lesions of the Acetabulum and Femoral Neck | JBJS Case Connect. | 10.2106/JBJS.CC.19.00026 | 31821201 |
| 41 | Papachristos et al. | 3 | 2019 | UK | Treatment of Incarcerated Impaction of Acetabular Fractures With Concomitant Osteochondral Femoral Head Fractures by the Use of a Posterior Wall Osteotomy and Surgical Hip Dislocation: A Novel Technique | Technique | adult | trauma | Acetabular fracture | ORIF | J Am Acad Orthop Surg. | 10.5435/JAAOS-D-18-00789 | 31246642 |
| 42 | Masquijo et al. | 5 | 2019 | Argentina | Treatment of Slipped Capital Femoral Epiphysis With the Modified Dunn Procedure: A Multicenter Study | case series | Pediatric | non-trauma | SCFE | modified Dunn procedure | J Pediatr Orthop. | 10.1097/BPO.0000000000000936 | 28106675 |
| 43 | Liu et al. | 7 | 2019 | China | Modified trapdoor procedures by surgical dislocation approach to treat chondroblastoma of the femoral head | case series | mixed | non-trauma | femoral head osteochondroma | curettage, and bone grafting | Bone Joint J. | 10.1302/0301-620X.101B6.BJJ-2018-1599.R1 | 31154843 |
| 44 | Leunig et al. | 3 | 2019 | Germany and Switzerland | Findings and Management of the Rare Caput Flexum Deformity of the Hip: A Case Report | case report | Pediatric | non-trauma | Caput Flexum Deformity of the Hip | anterior open wedge femoral neck osteotomy | JBJS Case Connect. | 10.2106/JBJS.CC.18.00321 | 31441832 |
| 45 | Kim et al. | 3 | 2019 | South Korea | Anterolateral Approach in the Treatment of Femoroacetabular Impingement of the Hip | case series | adult | non-trauma | FAI | osteochondroplasty | Clin Orthop Surg. | 10.4055/cios.2019.11.3.337 | 31475056 |
| 46 | Haefeli et al. | 5 | 2019 | Switzerland | Subchondral drilling for chondral flaps reduces the risk of total hip arthroplasty in femoroacetabular impingement surgery at minimum five years follow-up | case series | adult | non-trauma | FAI | Subchondral drilling for acetabular chondral flaps | Hip Int. | 10.1177/1120700018781807 | 29974796 |
| 47 | Ahmad et al. | 4 | 2019 | Germany and Switzerland | Surgical hip dislocation is more powerful than arthroscopy for achieving high degrees of acetabular correction in pincer type impingement | cohort | adult | non-trauma | FAI | according to the pathology | Orthop Traumatol Surg Res. | 10.1016/j.otsr.2019.08.009 | 31564633 |
| 48 | Trikha et al. | 4 | 2018 | India | Midterm results of trochanteric flip osteotomy technique for management of fractures around the hip | case series | adult | trauma | fracture around the hip | according to the injury present | Hip Int. | 10.5301/hipint.5000539 | 29027187 |
| 49 | Rego et al. | 6 | 2018 | Portugal and Germany | Arthroscopic versus open treatment of cam-type femoro-acetabular impingement: retrospective cohort clinical study | cohort | adult | non-trauma | FAI | osteochondroplasty | Int Orthop. | 10.1007/s00264-017-3735-4 | 29299653 |
| 50 | Hufeland et al. | 4 | 2018 | Germany | Long-term outcome of pigmented villonodular synovitis of the hip after joint preserving therapy | case series | adult | non-trauma | PVNS | synovectomy | Arch Orthop Trauma Surg. | 10.1007/s00402-017-2864-y | 29282525 |
| 51 | Ahmad et al. | 7 | 2018 | Pakistan | Capsular arthroplasty for neglected developmental dysplasia of hip | case series | mixed | non-trauma | neglected DDH | Colonna interposition arthroplasty (capsular arthroplasty) | J Orthop Surg (Hong Kong) | 10.1177/2309499018777888 | 29871541 |
| 52 | ZurmUhle et al. | 7 | 2017 | Switzerland | Periacetabular Osteotomy Provides Higher Survivorship Than Rim Trimming for Acetabular Retroversion | cohort | adult | non-trauma | FAI | acetabular rim trimming or PAO | Clin Orthop Relat Res. | 10.1007/s11999-016-5177-5 | 27921206 |
| 53 | Yao et al. | 7 | 2017 | China | Clinical reports of surgical dislocation of the hip with sequestrum clearance and impacting bone graft for grade IIIA-IIIB aseptic necrosis of femoral head (ANFH) patients | case series | adult | non-trauma | AVN | sequestrum clearance and impacting bone graft | Oncotarget. | 10.18632/oncotarget.15095 | 28177916 |
| 54 | Vaishya et al. | 4 | 2017 | India | Isolated acetabular osteochondroma of the hip | case report | adult | non-trauma | acetabular osteochondroma | excision and grafting | Acta Orthop Traumatol Turc. | 10.1016/j.aott.2015.12.001 | 28869065 |
| 55 | Schweitzer et al. | 5 | 2017 | Chile | Surgical dislocation of the hip without trochanteric osteotomy | case series | adult | non-trauma | Tumors and retained bullet | tumor resection and bullet extraction | J Orthop Surg (Hong Kong). | 10.1177/2309499016684414 | 29185379 |
| 56 | Haefeli et al. | 5 | 2017 | Switzerland and USA | Patients undergoing surgical hip dislocation for the treatment of acetabular fractures show favorable long-term outcome | case series | adult | trauma | acetabular fracture | ORIF | Bone Joint J. | 10.1302/0301-620X.99B4.37681 | 28385941 |
| 57 | Haefeli et al. | 5 | 2017 | Switzerland | What Are the Risk Factors for Revision Surgery After Hip Arthroscopy for Femoroacetabular Impingement at 7-year Follow up? | cohort | adult | non-trauma | FAI | OSTEOCHONDROPLASTY | ,Clin Orthop Relat Res. | 10.1007/s11999-016-5115-6 | 27718121 |
| 58 | Guindani et al. | 5 | 2017 | Italy and Germany | Surgical dislocation for pediatric and adolescent hip deformity: clinical and radiographical results at 3آ years follow-up | case series | mixed | non-trauma | Legg-Calvé-Perthes disease, SCFE, FAI, AVN, multiple hereditary exostoses and PVNS | ACCORDING TO THE CONDITION | Arch Orthop Trauma Surg. | 10.1007/s00402-017-2644-8 | 28197752 |
| 59 | Anwander et al. | 4 | 2017 | Switzerland | Labral Reattachment in Femoroacetabular Impingement Surgery Results in Increased 10-year Survivorship Compared With Resection | cohort | adult | non-trauma | FAI | osteoplasty and acetabular rim trimming | Clin Orthop Relat Res. | 10.1007/s11999-016-5114-7 | 27744594 |
| 60 | Won et al. | 5 | 2016 | South Korea | Osteochondral Autograft from the Ipsilateral Femoral Head by Surgical Dislocation for Treatment of Femoral Head Fracture Dislocation: A Case Report | case report | adult | trauma | FHF | Osteochondral Autograft from the Ipsilateral Femoral Head | Yonsei Med J. | 10.3349/ymj.2016.57.6.1527 | 27593886 |
| 61 | Van Nortwick et al. | 3 | 2016 | USA | Adolescent Hip Fracture-Dislocation: Transphyseal Fracture with Posterior Dislocation of the Proximal Femoral Epiphysis: A Case Report | case report | Pediatric | trauma | Transphyseal Fracture | ORIF | JBJS Case Connect. | 10.2106/JBJS.CC.15.00271 | 29252639 |
| 62 | Sorel et al. | 6 | 2016 | Netherlands | Surgical hip dislocation according to Ganz for excision of osteochondromas in patients with multiple hereditary exostoses | case series | mixed | non-trauma | osteochondroma OF femoral neck | resection | Bone Joint J. | 10.1302/0301-620X.98B2.36521 | 26850433 |
| 63 | Snoap et al. | 3 | 2016 | USA | Management of a Combined Femoral Head and Neck Fracture: A Case Report | case report | adult | trauma | FHF | ORIF | JBJS Case Connect. | 10.2106/JBJS.CC.16.00066 | 29252742 |
| 64 | Rüdiger et al. | 4 | 2016 | switzerland | Trans-foveal approach for curettage and bone grafting of a giant cell tumor in the femoral head | case report | adult | non-trauma | GCT femoral head | Curettage and iliac bone graft | Hip Int. | 10.5301/hipint.5000398 | 27740684 |
| 65 | Probe R. | 1 | 2016 | USA | Surgical Dislocation of the Hip in the Treatment of Complex Osteochondral Injury to the Femoral Head | Technique | adult | trauma | FHF with Osteochondral Injury | ORIF | J Orthop Trauma. | 10.1097/BOT.0000000000000608 | 27441926 |
| 66 | Novais et al. | 4 | 2016 | USA | Surgical Hip Dislocation for the Treatment of Intra-Articular Injuries and Hip Instability Following Traumatic Posterior Dislocation in Children and Adolescents | case series | Pediatric | trauma | hip dislocation and fractures | according to the intraoperative pathology and injury | J Pediatr Orthop. | 10.1097/BPO.0000000000000527 | 25985375 |
| 67 | Maqungo et al. | 7 | 2016 | South Afirca and Switzerland | Surgical hip dislocation for removal of retained intra-articular bullets | case series | adult | non-trauma | retained intra-articular bullets | extraction | Injury. | 10.1016/j.injury.2016.06.020 | 27375011 |
| 68 | Louahem et al. | 6 | 2016 | France | Mosaicplasty for femoral osteochondritis dissecans | case series | adult | non-trauma | OCD | mosaicplasty | Orthop Traumatol Surg Res. | 10.1016/j.otsr.2015.12.013 | 26896409 |
| 69 | Inan et al. | 3 | 2016 | Turkey | Treatment of mixed type femoroacetabular impingement using safe surgical hip dislocation in adults | case series | adult | non-trauma | FAI | according to the type of deformity present | Eklem Hastalik Cerrahisi | 10.5606/ehc.2016.32 | 27902171 |
| 70 | Hanke et al. | 5 | 2016 | Switzerland | Hips With Protrusio Acetabuli Are at Increased Risk for Failure After Femoroacetabular Impingement Surgery: A 10-year Follow up | case series | adult | non-trauma | FAI | circumferential rim trimming | Clin Orthop Relat Res. | 10.1007/s11999-016-4918-9 | 27278677 |
| 71 | Eid MA. | 1 | 2016 | Egypt | Hip preservation surgery for adolescents and young adults with Post-Perthes Sequelae | case series | adult | non-trauma | Post-Perthes Sequelae | Osteochondroplasty at the femoral head-neck junction and relative lengthening | Acta Orthop Belg. | 82(4):821-828.,Eid MA,Acta Orthop Belg,2016,29-11-17,,, | 29182124 |
| 72 | Balakumar and Madan | 2 | 2016 | UK | Late correction of neck deformity in healed severe slipped capital femoral epiphysis: short-term clinical outcomes | case series | mixed | non-trauma | SCFE | patients with an open physis underwent modified Dunn capital realignment and patients with a closed physis underwent a corrective neck osteotomy | Hip Int. | 10.5301/hipint.5000347 | 27229167 |
| 73 | Abdelazeem et al. | 5 | 2016 | Egypt | The anatomical reduction of a moderate or severe stable slipped capital femoral epiphysis by modified Dunn sub capital osteotomy using the Ganz approach: functional and radiological outcomes | case series | Pediatric | non-trauma | SCFE | open reduction and internal fixation using an intracapsular osteotomy | Bone Joint J. | 10.1302/0301-620X.98B9.37071 | 27587533 |
| 75 | Podeszwa et al. | 4 | 2015 | USA | Surgical Hip Dislocation is Safe and Effective Following Acute Traumatic Hip Instability in the Adolescent | case series | mixed | trauma | hip dislocation and fractures | procedures were performed based on intraarticular findings | J Pediatr Orthop. | 10.1097/BPO.0000000000000316 | 25197945 |
| 76 | Mladenovi et al. | 6 | 2015 | Serbia | Surgical dislocation of the hip in patients with femoroacetabular impingement: Surgical techniques and our experience | case series | adult | non-trauma | FAI | OSTEOCHONDROPLASTY | Vojnosanit Pregl. | 10.2298/vsp131207097m | 26731975 |
| 77 | Masse et al. | 5 | 2015 | Italy and Switzerland | Surgical hip dislocation is a reliable approach for treatment of femoral head fractures | case series | adult | trauma | FHF | ORIF | Clin Orthop Relat Res. | 10.1007/s11999-015-4352-4 | 25995178 |
| 78 | Gavaskar and Tummala | 2 | 2015 | India | Ganz Surgical Dislocation of the Hip Is a Safe Technique for Operative Treatment of Pipkin Fractures. Results of a Prospective Trial | case series | adult | trauma | FHF | ORIF | J Orthop Trauma. | 10.1097/BOT.0000000000000399 | 26595593 |
| 79 | Hingsammer et al. | 5 | 2015 | Switzerland | Is acetabular osteoplasty always required in mixed impingement? | case series | adult | non-trauma | FAI | osteochondroplasty with rim tri | Eur J Orthop Surg Traumatol. | 10.1007/s00590-014-1507-z | 25047732 |
| 80 | Gagala et al. | 3 | 2015 | Poland | Mosaicplasty for the treatment of osteochondritis dissecans following Legg-Calv-Perthes disease: a case report and literature review | case report | adult | non-trauma | OCD | Osteochondral autologous transfer (OATS) | Acta Orthop Traumatol Turc. | 10.3944/AOTT.2015.13.0012 | 26511699 |
| 81 | Clohisy et al. | 5 | 2015 | USA | Does surgical hip dislocation and periacetabular osteotomy improve pain in patients with Perthes-like deformities and acetabular dysplasia? | case series | adult | non-trauma | FAI | PAO | Clin Orthop Relat Res. | 10.1007/s11999-014-4115-7 | 25560960 |
| 82 | Bilge et al. | 5 | 2015 | Turkey and USA | Treatment of osteonecrosis of the femoral head with focal anatomic-resurfacing implantation (HemiCAP): preliminary results of an alternative option | case series | adult | non-trauma | AVN | A focal resurfacing implant | J Orthop Surg Res. | 10.1186/s13018-015-0199-3 | 25924980 |
| 83 | Beaul et al. | 4 | 2015 | Canada | Surgical Dislocation of the Hip for the Treatment of Pre-Arthritic Hip Disease | case series | mixed | non-trauma | FAI | Management according to the present pathology | J Arthroplasty. | 10.1016/j.arth.2015.04.009 | 25913231 |
| 84 | Steppacher et al. | 5 | 2014 | Switzerland | Surgical hip dislocation for treatment of femoroacetabular impingement: factors predicting 5-year survivorship | case series | adult | non-trauma | FAI | osteoplasty, and labral reattachment | Clin Orthop Relat Res. | 10.1007/s11999-013-3268-0 | 24014286 |
| 84 | Steppacher et al. | 5 | 2015 | Switzerland | Eighty percent of patients with surgical hip dislocation for femoroacetabular impingement have a good clinical result without osteoarthritis progression at 10 years | case series | adult | non-trauma | FAI | resection of a cam lesion and/or acetabular rim trimming including labral reattachment | Clin Orthop Relat Res. | 10.1007/s11999-014-4025-8 | 25367110 |
| 85 | Novais et al. | 6 | 2014 | USA | Open surgical treatment of femoroacetabular impingement in adolescent athletes: preliminary report on improvement of physical activity level | case series | mixed | non-trauma | FAI | osteochondroplasty | J Pediatr Orthop. | 10.1097/BPO.0000000000000093 | 24172673 |
| 86 | Mladenovi et al. | 7 | 2014 | Serbia | Early clinical results of surgical treatment of patients with femoroacetabular impingement | case series | adult | non-trauma | FAI | osteochondroplasty | Srp Arh Celok Lek. | 10.2298/sarh1406325m | 25033589 |
| 87 | Meulenkamp et al. | 3 | 2014 | USA | Viability assessment of the chondral flap in patients with cam-type femoroacetabular impingement: a preliminary report | case series | adult | non-trauma | FAI | osteochondroplasty | Can J Surg. | 10.1503/cjs.003513 | 24461226 |
| 88 | McLawhorn et al. | 7 | 2014 | USA | Fibromyxoid pseudotumor of the ligamentum teres treated with fresh osteochondral allograft | case report | adult | non-trauma | Fibromyxoid pseudotumor of the ligamentum teres | standard osteochondroplasty of the head-neck junction, fresh osteochondral allograft | Skeletal Radiol. | 10.1007/s00256-013-1752-y | 24150832 |
| 89 | Maini et al. | 6 | 2014 | India | Surgical dislocation of the hip for reduction of acetabular fracture and evaluation of chondral damage | case series | adult | trauma | acetabular fracture | ORIF | J Orthop Surg (Hong Kong). | 10.1177/230949901402200107 | 24781607 |
| 90 | Khanna et al. | 7 | 2014 | Canada | Cartilage restoration of the hip using fresh osteochondral allograft: resurfacing the potholes | case series | adult | non-trauma | AVN and OCD | Cartilage restoration using fresh osteochondral allograft | Bone Joint J. | 10.1302/0301-620X.96B11.34734 | 25381401 |
| 91 | Gagala et al. | 3 | 2014 | Poland | Fixation of femoral head fractures with autologous osteochondral transfer (mosaicplasty) | case report | adult | trauma | FHF | Autologous Osteochondral Transfer (Mosaicplasty) | J Orthop Trauma. | 10.1097/BOT.0000000000000079 | 24534892 |
| 92 | Botser et al. | 6 | 2014 | USA | Open surgical dislocation versus arthroscopic treatment of femoroacetabular impingement | cohort | adult | non-trauma | FAI | According to the pathology | Am J Orthop (Belle Mead NJ). | 43(5):209-14.,Botser IB,Am J Orthop (Belle Mead NJ),2014,20-05-14,,, | 24839626 |
| 93 | Bali et al. | 4 | 2014 | Canada | Sub capital osteotomy of the femoral neck for patients with healed slipped capital femoral epiphysis | case series | mixed | non-trauma | SCFE | a sub capital intracapsular osteotomy of the femoral neck | Bone Joint J. | 10.1302/0301-620X.96B11.33519 | 25371454 |
| 94 | Abolghasemian et al. | 6 | 2014 | Iran and Canada | Hips with synovial chondromatosis may display the features of femoroacetabular impingement | case series | adult | non-trauma | synovial chondromatosis | excision of the synovial chondromatosis loose bodies, and reshaping of the femoral head-neck junction | J Bone Joint Surg Am. | 10.2106/JBJS.L.01550 | 24430420 |
| 95 | Zingg et al. | 6 | 2013 | Switzerland | Surgical hip dislocation versus hip arthroscopy for femoroacetabular impingement: clinical and morphological short-term results | cohort | adult | non-trauma | FAI | According to the pathology | Arch Orthop Trauma Surg. | 10.1007/s00402-012-1616-2 | 23064993 |
| 96 | Sink et al. | 5 | 2013 | USA | Results of treatment of femoroacetabular impingement in adolescents with a surgical hip dislocation approach | case series | mixed | non-trauma | FAI | surgical hip dislocation for FAI | Clin Orthop Relat Res. | 10.1007/s11999-013-3004-9 | 23653097 |
| 97 | Masse et al. | 5 | 2013 | Italy | Surgical dislocation technique for the treatment of acetabular fractures | case series | adult | trauma | acetabular fracture | ORIF | Clin Orthop Relat Res. | 10.1007/s11999-013-3228-8 | 24002867 |
| 98 | Madan et al. | 4 | 2013 | UK | The treatment of severe slipped capital femoral epiphysis via the Ganz surgical dislocation and anatomical reduction: a prospective study | case series | mixed | non-trauma | FAI after SCFE | modified Dunn procedure | Bone Joint J. | 10.1302/0301-620X.95B3.30113 | 23450032 |
| 99 | Kosashvili et al. | 6 | 2013 | Israel | Fresh-stored osteochondral allografts for the treatment of femoral head defects: surgical technique and preliminary results | case series | adult | non-trauma | femoral head defect (AVN-OCD) | Fresh-stored osteochondral allografts | Int Orthop. | 10.1007/s00264-013-1868-7 | 23553116 |
| 100 | Gunel et al. | 3 | 2013 | Turkey | Long-term follow-up of a hip joint osteoblastoma after intralesional curettage and cement packing: a case report | case report | adult | non-trauma | osteoblastoma of the acetabulum | polymethyl-methacrylate was packed inside the gap of the acetabular site after intralesional wide curettage | Acta Orthop Traumatol Turc. | 10.3944/aott.2013.2734 | 23748623 |
| 101 | Domb et al. | 4 | 2013 | USA | Surgical dislocation of the hip versus arthroscopic treatment of femoroacetabular impingement: a prospective matched-pair study with average 2-year follow-up | cohort | adult | non-trauma | FAI | FAI treated with surgical dislocation or arthroscopy | Arthroscopy. | 10.1016/j.arthro.2013.06.010 | 23992988 |
| 102 | Costa Rocha et al. | 5 | 2013 | Portugal | Circumferential reconstruction of severe acetabular labral damage using hamstring allograft: surgical technique and case series | case series | adult | non-trauma | FAI with labral damage | surgical hip dislocation to address femoroacetabular impingement with rim trimming, femoral osteochondroplasty, and labral reconstruction | Hip Int. | 10.5301/HIP.2013.11662 | 24318364 |
| 103 | Buchler et al. | 6 | 2013 | Switzerland | Arthroscopic versus open cam resection in the treatment of femoroacetabular impingement | cohort | adult | non-trauma | FAI | managing cam or mixed type femoroacetabular impingement (FAI) accordingly | Arthroscopy. | 10.1016/j.arthro.2012.12.009 | 23395249 |
| 104 | Anderson et al. | 4 | 2013 | USA | Sub capital correction osteotomy for malunited slipped capital femoral epiphysis | case series | adult | non-trauma | FAI after SCFE | patients with closed femoral physis and symptomatic femoroacetabular impingement from malunited SCFE treated with surgical dislocation and SCO | J Pediatr Orthop. | 10.1097/BPO.0b013e31827d7e06 | 23653020 |
| 105 | Walker et al. | 4 | 2012 | USA | Preliminary pain and function after labral reconstruction during femoroacetabular impingement surgery | case series | adult | non-trauma | FAI | labral reconstruction | Clin Orthop Relat Res. | 10.1007/s11999-012-2506-1 | 22864618 |
| 106 | Shore et al. | 4 | 2012 | USA | Low early failure rates using a surgical dislocation approach in healed Legg-Calv-Perthes disease | case series | mixed | non-trauma | Legg-Calvé-Perthes disease | femoral head-neck osteochondroplasty | Clin Orthop Relat Res. | 10.1007/s11999-011-2187-1 | 22125243 |
| 107 | Ross et al. | 5 | 2012 | USA | Intraarticular abnormalities in residual Perthes and Perthes-like hip deformities | case series | mixed | non-trauma | Legg-Calvé-Perthes disease | any labral or cartilage lesions were addressed accordingly | Clin Orthop Relat Res. | 10.1007/s11999-012-2375-7 | 22569718 |
| 108 | Naal et al. | 5 | 2012 | Switzerland | Midterm results of surgical hip dislocation for the treatment of femoroacetabular impingement | case series | adult | non-trauma | FAI | Osteochondroplasty | Am J Sports Med. | 10.1177/0363546512445884 | 22556199 |
| 109 | Masse et al. | 6 | 2012 | Italy | Surgical hip dislocation for anatomic reorientation of slipped capital femoral epiphysis: preliminary results | case series | mixed | non-trauma | SCFE | sub capital re-orientation | Hip Int. | 10.5301/HIP.2012.9208 | 22505180 |
| 110 | Manner et al. | 4 | 2012 | Switzerland | Potential contribution of femoroacetabular impingement to recurrent traumatic hip dislocation | case report | adult | trauma | recurrent traumatic hip dislocation | Refixation of the peri-osteolabral complex, capsular closure , anterior femoral osteochondroplasty combined with debridement of the anterior chondral flap | J Pediatr Orthop B. | 10.1097/BPB.0b013e328357bf04 | 22871962 |
| 111 | Li et al. | 4 | 2012 | USA | Operative treatment of femoral neck osteochondroma through a digastric approach in a pediatric patient: a case report and review of the literature | case report | Pediatric | non-trauma | femoral neck osteochondroma | Excision | J Pediatr Orthop B. | 10.1097/BPB.0b013e3283524bc3 | 22422006 |
| 112 | Leunig et al. | 5 | 2012 | Switzerland | Surgical technique: Second-generation bone marrow stimulation via surgical dislocation to treat hip cartilage lesions | case series | adult | non-trauma | femoral head cartilage lesion | Second-generation Bone Marrow Stimulation+ GRAFTING | Clin Orthop Relat Res. | 10.1007/s11999-012-2466-5 | 22773396 |
| 113 | Ganz et al. | 5 | 2012 | Switzerland | Surgical technique: The capsular arthroplasty: a useful but abandoned procedure for young patients with developmental dysplasia of the hip | case series | mixed | non-trauma | DDH | Capsular Arthroplasty | Clin Orthop Relat Res. | 10.1007/s11999-012-2444-y | 22733187 |
| 114 | Emre et al.. | 5 | 2012 | Turkey | Mosaicplasty for the treatment of the osteochondral lesion in the femoral head | case report | adult | non-trauma | osteochondral defect | Mosaicplasty | Bull NYU Hosp Jt Dis. |  | 23267459 |
| 115 | Albers et al. | 5 | 2012 | Switzerland | "Joint-preserving surgery improves pain range of motion and abductor strength after Legg-Calv-Perthes disease | case series | mixed | non-trauma | Legg-Calvé-Perthes disease | PAO and intertrochanteric osteotomy | Clin Orthop Relat Res. | 10.1007/s11999-012-2345-0 | 22528379 |
| 116 | Nepple et al. | 3 | 2011 | USA | Treatment of posttraumatic labral interposition with surgical hip dislocation and labral repair | case report | Pediatric | trauma | POSTTRAUMATIC LABRAL INTERPOSITION | LABRAL REPAIR | Iowa Orthop J. |  | 22096440 |
| 117 | Naalet al. | 4 | 2011 | Switzerland | Surgical hip dislocation for the treatment of femoroacetabular impingement in high-level athletes | case series | adult | non-trauma | FAI | Management according to the type (cam- pincer or mixed-type FAI) | Am J Sports Med. | 10.1177/0363546510387263 | 21173196 |
| 118 | Leunig and Ganz | 2 | 2011 | Switzerland | Relative neck lengthening and intramarital osteotomy for severe Perthes and Perthes-like deformities | case series | mixed | non-trauma | Legg-Calvé-Perthes disease and Perthes like deformity | Relative neck lengthening and intra-capital osteotomy | Bull NYU Hosp Jt Dis. |  | 22035488 |
| 119 | Kempthorne et al. | 4 | 2011 | New Zeland | Surgical dislocation of the hip and the management of femoroacetabular impingement: results of the Christchurch experience | case series | adult | non-trauma | FAI | Osteochondroplasty | ANZ J Surg. | 10.1111/j.1445-2197.2010.05489.x | 22295348 |
| 120 | Jager et al. | 7 | 2011 | Germany | Surgical hip dislocation in symptomatic cam femoroacetabular impingement: what matters in early good results? | case series | adult | non-trauma | FAI | Osteochondroplasty | Eur J Med Res. | 10.1186/2047-783x-16-5-217 | 21719395 |
| 121 | Huber et al. | 5 | 2011 | Switzerland | Adolescent slipped capital femoral epiphysis treated by a modified Dunn osteotomy with surgical hip dislocation | case series | mixed | non-trauma | SCFE | modified Dunn osteotomy | J Bone Joint Surg Br. | 10.1302/0301-620X.93B6.25849 | 21586786 |
| 122 | Girard et al. | 4 | 2011 | France | Osteochondral mosaicplasty of the femoral head | case series | adult | non-trauma | femoral head Osteochondral lesion | osteochondral autograft transplant | Hip Int. | 10.5301/HIP.2011.8659 | 21948031 |
| 123 | Botser et al. | 4 | 2011 | USA | Open surgical dislocation versus arthroscopy for femoroacetabular impingement: a comparison of clinical outcomes | cohort | adult | non-trauma | FAI | open surgical dislocation, arthroscopic, and combined approaches for Femoroacetabular Impingement | Arthroscopy. | 10.1016/j.arthro.2010.11.008 | 21266277 |
| 124 | Bedi et al. | 4 | 2011 | USA | Radiographic comparison of surgical hip dislocation and hip arthroscopy for treatment of cam deformity in femoroacetabular impingement | cohort | adult | non-trauma | FAI | osteoplasty, and labral debridement or refixation | Am J Sports Med. | 10.1177/0363546511412734 | 21709028 |
| 125 | Beck and Buchler | 2 | 2011 | Switzerland | Prevalence and impact of pain at the greater trochanter after open surgery for the treatment of femoro-acetabular impingement | case series | adult | non-trauma | FAI | Osteochondroplasty | J Bone Joint Surg Am. | 10.2106/JBJS.J.01718 | 21543692 |
| 126 | Tannast et al. | 6 | 2010 | Switzerland | Surgical dislocation of the hip for the fixation of acetabular fractures | case series | adult | trauma | acetabulum fracture | ORIF | J Bone Joint Surg Br. | 10.1302/0301-620X.92B6.22994 | 20513883 |
| 127 | Slongo et al. | 4 | 2010 | Switzerland | Treatment of slipped capital femoral epiphysis with a modified Dunn procedure | case series | mixed | non-trauma | SCFE | Modified Dunn Procedure | J Bone Joint Surg Am. | 10.2106/JBJS.I.01385 | 21159990 |
| 128 | Sink et al. | 4 | 2010 | USA | Acetabular cartilage and labral damage observed during surgical hip dislocation for stable slipped capital femoral epiphysis | case series | NR | non-trauma | SCFE | epiphyseal reduction | J Pediatr Orthop. | 10.1097/BPO.0b013e3181c6b37a | 20032738 |
| 129 | Peters et al. | 4 | 2010 | USA | Open treatment of femoroacetabular impingement is associated with clinical improvement and low complication rate at short-term follow up | case series | adult | non-trauma | FAI | Osteochondroplasty, Labral reconstruction, PAO (management options were performed according to the pathology) | Clin Orthop Relat Res. | 10.1007/s11999-009-1152-8 | 19885709 |
| 130 | Naranje et al. | 5 | 2010 | India | Digastric trochanteric flip osteotomy and surgical dislocation of hip in the management of acetabular fractures | case series | adult | trauma | acetabulum fracture | ORIF | Arch Orthop Trauma Surg. | 10.1007/s00402-009-0873-1 | 19373481 |
| 131 | Keel et al. | 4 | 2010 | Switzerland | Surgical dislocation of the hip for a locked traumatic posterior dislocation with associated femoral neck and acetabular fractures | case report | adult | trauma | acetabulum fracture | ORIF | J Bone Joint Surg Br. | 10.1302/0301-620X.92B3.23016 | 20190319 |
| 132 | Bastian et al. | 5 | 2010 | Switzerland | Surgical hip dislocation for osteochondral transplantation as a salvage procedure for a femoral head impaction fracture | case report | adult | trauma | femoral head impaction fracture | Osteochondral Transplantation | J Orthop Trauma. | 10.1097/BOT.0b013e3181dfbb52 | 21076245 |
| 133 | Anderson et al. | 4 | 2010 | USA | Sequelae of Perthes disease: treatment with surgical hip dislocation and relative femoral neck lengthening | case series | mixed | non-trauma | Sequelae OF Legg-Calvé-Perthes disease | Relative Femoral Neck Lengthening | J Pediatr Orthop. | 10.1097/BPO.0b013e3181fcbaaf | 21102198 |
| 134 | Yun et al. | 3 | 2009 | south Korea | Treatment of femoroacetabular impingement with surgical dislocation | case series | adult | non-trauma | FAI | Osteochondroplasty | Clin Orthop Surg. | 10.4055/cios.2009.1.3.146 | 19885050 |
| 135 | Van Stralen et al. | 4 | 2009 | The Netherlands | Partial resurfacing with varus osteotomy for an osteochondral defect of the femoral head | case report | adult | non-trauma | femoral head osteochondral defect | Partial resurfacing with varus osteotomy | Hip Int. | 10.1177/112070000901900113 | 19455506 |
| 136 | Solberg et al. | 3 | 2009 | USA | Use of a trochanteric flip osteotomy improves outcomes in Pipkin IV fractures | case series | adult | trauma | FHF | ORIF | Clin Orthop Relat Res. | 10.1007/s11999-008-0505-z | 18800211 |
| 137 | Sierra and Trousdale | 2 | 2009 | USA | Labral reconstruction using the ligamentum teres capitis: report of a new technique | case report | adult | non-trauma | FAI with labral damage | Osteochondroplasty and Labral Reconstruction Using the Ligamentum Teres | Clin Orthop Relat Res. | 10.1007/s11999-008-0633-5 | 19048354 |
| 138 | Shin et al. | 7 | 2009 | south Korea | Application of Ganz surgical hip dislocation approach in pediatric hip diseases | case series | Pediatric | non-trauma | various conditions | Management was tailored to each condition | Clin Orthop Surg. | 10.4055/cios.2009.1.3.132 | 19885048 |
| 139 | Shin et al. | 6 | 2009 | south Korea | Unusual osseocartilaginous prominence or bump causing femoroacetabular impingement after septic arthritis of the hip: a report of 2 cases in preadolescence | case report | Pediatric | non-trauma | FAI | Osteochondroplasty | J Pediatr Orthop. | 10.1097/BPO.0b013e3181aa2465 | 19568017 |
| 140 | Rebello et al. | 4 | 2009 | USA | Surgical dislocation in the management of pediatric and adolescent hip deformity | case series | mixed | non-trauma | hip deformities | Osteoplasty and intertrochanteric osteotomy | Clin Orthop Relat Res. | 10.1007/s11999-008-0591-y | 19002743 |
| 141 | Padhy et al. | 6 | 2009 | south Korea | Femoroacetabular impingement due to synovial chondromatosis of the hip joint | case report | adult | non-trauma | synovial chondromatosis | Synovectomy | Orthopedics. | 10.3928/01477447-20091020-30 | 19968228 |
| 142 | Nakamura et al. | 3 | 2009 | Japan | Synovial osteochondromatosis of the hip treated through a surgical dislocation | case report | adult | non-trauma | synovial chondromatosis | Synovectomy | J Arthroplasty. | 10.1016/j.arth.2008.09.004 | 18835688 |
| 143 | Khan et al. | 3 | 2009 | UK | "Hip resurfacing through a modified anterolateral approach as compared with the Ganz trochanteric flip osteotomy: a two year follow-up study | cohort | adult | non-trauma | OA | hip resurfacing | Hip Int. | 10.1177/112070000901900407 | 20041380 |
| 144 | Jellicoe et al. | 4 | 2009 | Canada | Surgical hip dislocation for removal of intraarticular exostoses: report of two cases | case report | Pediatric | non-trauma | exostoses | excision | J Pediatr Orthop. | 10.1097/BPO.0b013e3181a56b4f | 19461371 |
| 145 | Graves and Mast | 2 | 2009 | USA | Femoroacetabular impingement: do outcomes reliably improve with surgical dislocations? | case series | adult | non-trauma | FAI | Osteochondroplasty | Clin Orthop Relat Res. | 10.1007/s11999-008-0648-y | 19082680 |
| 146 | Beaule et al. | 3 | 2009 | Canada | Clinical experience of Ganz surgical dislocation approach for metal-on-metal hip resurfacing | case series | adult | non-trauma | OA, AVN, HIP DYSPLASIA, Perthes | Hip resurfacing | J Arthroplasty. | 10.1016/j.arth.2009.04.006 | 19553070 |
| 147 | Anderson et al. | 4 | 2009 | USA | Staged surgical dislocation and redirectional periacetabular osteotomy: a report of five cases | case report | adult | non-trauma | FAI | redirectional Peri-acetabular osteotomy | J Bone Joint Surg Am. | 10.2106/JBJS.H.00066 | 19797584 |
| 148 | Leunig et al. | 4 | 2007 | Switzerland | Sub capital correction osteotomy in slipped capital femoral epiphysis by means of surgical hip dislocation | case series | Pediatric | non-trauma | SCFE | Sub capital Correction Osteotomy | Oper Orthop Traumatol. | 10.1007/s00064-007-1213-7 | 17940736 |
| 149 | Beaule et al. | 3 | 2007 | Canada | Quality of life following femoral head-neck osteochondroplasty for femoroacetabular impingement | case series | adult | non-trauma | FAI | Osteochondroplasty | J Bone Joint Surg Am. | 10.2106/JBJS.F.00681 | 17403799 |
| 150 | Espinosa et al. | 5 | 2007 | Switzerland | Treatment of femoro-acetabular impingement: preliminary results of labral refixation. Surgical technique | case series | adult | non-trauma | FAI | Osteochondroplasty | J Bone Joint Surg Am. | 10.2106/JBJS.F.01123 | 17332124 |
| 151 | Spencer et al. | 3 | 2006 | USA | Early results of treatment of hip impingement syndrome in slipped capital femoral epiphysis and pistol grip deformity of the femoral head-neck junction using the surgical dislocation technique | case series | adult | non-trauma | FAI | Osteochondroplasty | ,J Pediatr Orthop. | 10.1097/01.bpo.0000217726.16417.74 | 16670535 |
| 152 | Schoeniger et al. | 5 | 2006 | Switzerland | Modified complete synovectomy prevents recurrence in synovial chondromatosis of the hip | case series | adult | non-trauma | synovial chondromatosis | Synovectomy | Clin Orthop Relat Res. | 10.1097/01.blo.0000229280.53109.d5 | 16760824 |
| 153 | Peters and Erickson | 2 | 2006 | USA | Treatment of femoro-acetabular impingement with surgical dislocation and debridement in young adults | case series | adult | non-trauma | FAI | osteochondroplasty | J Bone Joint Surg Am. | 10.2106/JBJS.E.00514 | 16882895 |
| 154 | Jager et al. | 3 | 2006 | Germany | Partial hemi-resurfacing of the hip joint, a new approach to treat local osteochondral defects? | Technique | adult | non-trauma | Femoral head chondral defect | Partial hemi-resurfacing using Hemi-cap | Biomed Tech (Berl). | 10.1515/BMT.2006.072 | 17155875 |
| 155 | Espinosa et al. | 5 | 2006 | Switzerland | Treatment of femoro-acetabular impingement: preliminary results of labral refixation | cohort | adult | non-trauma | FAI | Osteochondroplasty | J Bone Joint Surg Am. | 10.2106/JBJS.E.00290 | 16651565 |
| 156 | Gardner et al. | 6 | 2005 | USA | Surgical dislocation of the hip for fractures of the femoral head | Technique | adult | trauma | FHF | ORIF | J Orthop Trauma. | 19(5):334-42.,Gardner MJ,J Orthop Trauma,2005,14-05-05,,, | 15891543 |
| 157 | Haverkamp et al. | 3 | 2004 | The Netherlands | Acetabular reduction osteotomy using surgical dislocation of the hip joint for treatment of a malunited acetabular fracture | case report | adult | trauma | malunited acetabular fracture | Reduction osteotomy | Arch Orthop Trauma Surg. | 10.1007/s00402-004-0731-0 | 15340749 |
| 158 | Leunig et al. | 4 | 2003 | Switzerland | Traumatic labral avulsion from the stable rim: a constant pathology in displaced transverse acetabular fractures | case series | adult | trauma | acetabular fracture | ORIF | Arch Orthop Trauma Surg. | 10.1007/s00402-003-0575-z | 14574595 |
| 159 | Siebenrock et al. | 5 | 2002 | Switzerland | Surgical dislocation of the femoral head for joint debridement and accurate reduction of fractures of the acetabulum | case series | adult | trauma | acetabular fracture | ORIF | J Orthop Trauma. | 10.1097/00005131-200209000-00002 | 12352562 |
| 160 | Ganz et al. | 6 | 2001 | Switzerland | Surgical dislocation of the adult hip a technique with full access to the femoral head and acetabulum without the risk of avascular necrosis | case series | adult | non-trauma | various conditions | Management was tailored to each condition | J Bone Joint Surg Br. | 10.1302/0301-620x.83b8.11964 | 11764423 |

**References:**

1. Zhou C, Fan Y, Liang Y, Wei Z, Liu Y, Li W, Wei Q, Fang H, He W, Chen Z (2022) Clinical Outcome of Surgical Hip Dislocation Combined with Impacting Bone Grafts and Implanting Iliac Bone Flaps in the Treatment of Osteonecrosis of the Femoral Head: A Mid-term Retrospective Study. Orthop Surg 14 (6):1115-1125. doi:10.1111/os.13295

2. Yoon YC, Oh CW, Kim JW, Heo J, Song HK (2022) Safety of surgical hip dislocation in femoral head fracture and dislocation (FHFD) and avascular necrosis risk factor analysis of FHFD: midterm results confirmed by SPECT/CT and MRI. J Orthop Surg Res 17 (1):278. doi:10.1186/s13018-022-03160-y

3. Wang Y, Bian Y, Chen X, Qian W (2022) Surgical treatment of femoroacetabular impingement in a patient with Stickler syndrome: a case report. Ann Transl Med 10 (8):495. doi:10.21037/atm-21-4784

4. Tripathy S, Varghese P, Sethy SS, Agrawal K (2022) Safe surgical hip dislocation for acetabular osteoid osteoma excision. BMJ Case Rep 15 (2). doi:10.1136/bcr-2021-246025

5. Leibold CS, Vuillemin N, Buchler L, Siebenrock KA, Steppacher SD (2022) Surgical hip dislocation with relative femoral neck lengthening and retinacular soft-tissue flap for sequela of Legg-Calve-Perthes disease. Oper Orthop Traumatol 34 (5):352-360. doi:10.1007/s00064-022-00780-9

6. Laboudie P, Gauthier P, Kreviazuk C, Beaule PE (2022) Does labral treatment technique influence the outcome of FAI surgery? A matched-pair study of labral reconstruction versus repair and debridement with a follow-up of 10 years. J Hip Preserv Surg 9 (2):95-101. doi:10.1093/jhps/hnac017

7. Klosterman EL, Zacharias AJ, Dooley MS, Wilson NM, Turner EHG, Goodspeed DC, Spiker AM (2022) Treatment of Coxa Profunda With Open Surgical Hip Dislocation, Rim Resection, Cam Resection, and Labral Reconstruction. Arthrosc Tech 11 (8):e1499-e1508. doi:10.1016/j.eats.2022.03.042

8. Hosny H, Mousa S, Salama W (2022) Management of femoral head fracture by Ganz surgical dislocation of the hip. J Orthop Traumatol 23 (1):24. doi:10.1186/s10195-022-00643-w

9. Feroe AG, Hassan MM, Fourman MS, Anderson ME, Kim YJ (2022) Surgical Hip Dislocation for a Diagnostic Dilemma: Differentiating Synovial Chondromatosis and Pigmented Villonodular Synovitis. Iowa Orthop J 42 (1):263-265

10. Diaz-Dilernia F, Astore F, Buttaro M, Zanotti G (2022) Short-Term Results of Osteochondral Autologous Transfer and Femoral Neck Osteochondroplasty for the Treatment of Osteochondral Lesions of the Femoral Head and Concomitant Femoroacetabular Impingement Syndrome: A Case Series. Hip Pelvis 34 (3):177-184. doi:10.5371/hp.2022.34.3.177

11. Chen W, Li J, Guo W, Gao S, Wei Q, Li Z, He W (2022) Outcomes of surgical hip dislocation combined with bone graft for adolescents and younger adults with osteonecrosis of the femoral head: a case series and literature review. BMC Musculoskelet Disord 23 (1):499. doi:10.1186/s12891-022-05456-w

12. Chen MJ, Hollyer I, Pun SY, Bellino MJ (2022) Acetabular labral reconstruction with medial meniscal allograft: preliminary results of a new surgical technique. Eur J Orthop Surg Traumatol 32 (3):515-521. doi:10.1007/s00590-021-02986-2

13. Baraka MM, Hefny HM, Thakeb MF, Mahran MA, El Ghazawy AK, Fayyad TA (2022) Morscher's femoral neck lengthening osteotomy through surgical hip dislocation approach for preservation of Perthes and Perthes-like deformities. J Child Orthop 16 (1):5-18. doi:10.1177/18632521221080477

14. Ahern S, O'Sullivan MD, Clesham K, Wade A, Meleady E, Green C (2022) Clinical and radiological outcomes following surgical hip dislocation for paediatric hip pathologies, a prospective cohort study. Surgeon. doi:10.1016/j.surge.2022.09.005

15. Abo-Elsoud M, Sadek W, Salah-Eldeen M, Abosenna W (2022) Surgical hip dislocation for treatment of femoral head chondroblastoma: efficacy and safety. Int Orthop 46 (3):653-660. doi:10.1007/s00264-021-05264-2

16. Abdelnasser MK, Refai O, Farouk O (2022) Surgical hip dislocation in fixation of acetabular fractures: Extended indications and outcome. Injury 53 (2):539-545. doi:10.1016/j.injury.2021.09.054

17. Youngman TR, Wagner KJ, 3rd, Montanez B, Johnson BL, Wilson PL, Morris WZ, Sucato DJ, Podeszwa DA, Ellis HB, Jr. (2021) The Association of alpha Angle on Disease Severity in Adolescent Femoroacetabular Impingement. J Pediatr Orthop 41 (2):88-92. doi:10.1097/BPO.0000000000001703

18. Trikha V, Das S, Mittal S, Chowdhury B (2021) An unusual pattern of posterior wall fracture of acetabulum. Eur J Orthop Surg Traumatol 31 (4):643-650. doi:10.1007/s00590-020-02814-z

19. Steppacher SD, Meier MK, Albers CE, Tannast M, Siebenrock KA (2021) Acetabular Cartilage Thickness Differs Among Cam, Pincer, or Mixed-Type Femoroacetabular Impingement: A Descriptive Study Using In Vivo Ultrasonic Measurements During Surgical Hip Dislocation. Cartilage 13 (2_suppl):465S-475S. doi:10.1177/1947603521990879

20. Sarassa C, Carmona D, Vanegas Isaza D, Restrepo Rodriguez C, Herrera Torres AM (2021) Long-Term Results of Slipped Capital Femoral Epiphysis Treated with the Modified Dunn Procedure in a Colombian Cohort. Clin Orthop Surg 13 (3):415-422. doi:10.4055/cios19038

21. Risto O, Sandquist S, Lind S, Madan S (2021) Outcome after osteochondroplasty and relative neck lengthening for patients with healed Legg-Calve-Perthes disease: a retrospective cohort study of patients with hip-deformities treated with osteochondroplasty and relative neck lengthening. Hip Int 31 (3):417-423. doi:10.1177/1120700019896767

22. Rego P, Mascarenhas V, Mafra I, Oliveira F, Pinto P, Ganz R (2021) Femoral neck osteotomy in skeletally mature patients: surgical technique and midterm results. Int Orthop 45 (1):83-94. doi:10.1007/s00264-020-04822-4

23. Mundluru SN, Podeszwa D, Peck J, Sucato D, Johnston C, Kim H, Wilson P, Ellis H (2021) Acetabular Coverage May Affect Radiographic and Clinical Outcomes of Osteochondral Allograft Transplantation of Focal Femoral Head Lesions: A Case Series From a Single Institution. J Am Acad Orthop Surg Glob Res Rev 5 (10). doi:10.5435/JAAOSGlobal-D-20-00219

24. Lieberman EG, Pascual-Garrido C, Abu-Amer W, Nepple JJ, Shoenecker PL, Clohisy JC (2021) Patients With Symptomatic Sequelae of Slipped Capital Femoral Epiphysis Have Advanced Cartilage Wear at the Time of Surgical Intervention. J Pediatr Orthop 41 (6):e398-e403. doi:10.1097/BPO.0000000000001797

25. Khalifa AA, Refai O, Farouk O, Abdelnasser MK (2021) Management of femoral head fractures through surgical hip dislocation (SHD): a demanding but safe technique. Arch Orthop Trauma Surg 141 (10):1701-1710. doi:10.1007/s00402-020-03666-2

26. Fang S, Li H, Wang Y, Xu P, Sun H, Li S, Wei Z, Sun X (2021) Surgical hip dislocation for treatment of synovial chondromatosis of the hip. Int Orthop 45 (11):2819-2824. doi:10.1007/s00264-021-05045-x

27. De Mauro D, Rovere G, Smakaj A, Marino S, Ciolli G, Perna A, Battiato C, El Ezzo O, Liuzza F (2021) Gibson approach and surgical hip dislocation according to Ganz in the treatment of femoral head fractures. BMC Musculoskelet Disord 22 (Suppl 2):961. doi:10.1186/s12891-021-04800-w

28. Colares PGB, Carlos LMB, Ramos M, Campos CPS, Nascimento VDD, Cassiano JGM, Valente TM (2021) Intraoperative blood salvage in proximal femur epiphysiolysis surgical treatment with hip controlled dislocation technique: a case series study. Braz J Anesthesiol 71 (5):545-549. doi:10.1016/j.bjane.2021.02.061

29. Aprato A, Buzzone M, Di Benedetto P, Masse A (2021) Surgical hip dislocation vs arthroscopy for fixation of subfoveal femoral head fractures: A new technique for Pipkin type 1 fractures. Acta Biomed 92 (S3):e2021016. doi:10.23750/abm.v92iS3.11724

30. Xia T, Liu J, Shi L, Zhang C, Yao C, Shen J (2020) Comparison of surgical dislocation and impacting bone graft and surgical dislocation and rotational osteotomy for the treatment of ARCO III femoral head necrosis. Medicine (Baltimore) 99 (20):e20215. doi:10.1097/MD.0000000000020215

31. Steppacher SD, Sedlmayer R, Tannast M, Schmaranzer F, Siebenrock KA (2020) Surgical hip dislocation with femoral osteotomy and bone grafting prevents head collapse in hips with advanced necrosis. Hip Int 30 (4):398-406. doi:10.1177/1120700019856010

32. Shahien AA, Wegrzyn K, Tornetta P, 3rd, Kain MS (2020) Surgical Hip Dislocation for Small Posterior Wall Fracture After Hip Subluxation. J Orthop Trauma 34 Suppl 2:S19-S20. doi:10.1097/BOT.0000000000001833

33. Nepple JJ, Zaltz I, Larson CM, Beaule PE, Kim YJ, Millis MB, Sierra RJ, Clohisy JC, Group A (2020) Surgical Treatment of Femoroacetabular Impingement: Hip Arthroscopy Versus Surgical Hip Dislocation: A Propensity-Matched Analysis. J Bone Joint Surg Am 102 (Suppl 2):51-58. doi:10.2106/JBJS.20.00265

34. Leibold CS, Schmaranzer F, Siebenrock KA, Steppacher SD (2020) Femoral osteotomies for the treatment of avascular necrosis of the femoral head. Oper Orthop Traumatol 32 (2):116-126. doi:10.1007/s00064-019-00642-x

35. Lamplot JD, Schoenecker PL, Pascual-Garrido C, Nepple JJ, Clohisy JC (2020) Open Reduction and Internal Fixation for the Treatment of Symptomatic Osteochondritis Dissecans of the Femoral Head in Patients With Sequelae of Legg-Calve-Perthes Disease. J Pediatr Orthop 40 (3):120-128. doi:10.1097/BPO.0000000000001192

36. Hanke MS, Keel MJB, Cullmann JL, Siebenrock KA, Bastian JD (2020) Transfer of osteochondral shell autografts to salvage femoral head impaction injuries in hip trauma patients. Injury 51 (3):711-718. doi:10.1016/j.injury.2020.01.037

37. Hancioglu S, Tosyali HK, Erkan S, Yercan HS (2020) Clinical and radiological outcomes of chronic severe slipped capital femoral epiphysis patients treated by surgical dislocation and modified Dunn osteotomy: Case series. Jt Dis Relat Surg 31 (3):599-604. doi:10.5606/ehc.2020.75101

38. Gavaskar AS, Srinivasan P, Jeyakumar B, Raj RV, Sharath V, Narayan DA (2020) Surgical Dislocation or the Modified Heuter Anterior Approach for Pipkin I and II Femoral Head Fracture Dislocations. J Orthop Trauma 34 (12):626-631. doi:10.1097/BOT.0000000000001877

39. Faure PA, Zaltz I, Cote K, Pelet S, Forsythe C, Beaule PE, Belzile EL (2020) Morscher Osteotomy Through Surgical Dislocation Approach for True Femoral Neck Lengthening with Greater Trochanter Transposition. J Bone Joint Surg Am 102 (Suppl 2):66-72. doi:10.2106/JBJS.20.00405

40. Shannon BA, Dixit S, McCarthy EF, Levin AS (2019) Surgical Hip Dislocation Using the Modified Hardinge Approach for Excision of Osteocartilaginous Lesions of the Acetabulum and Femoral Neck in an Adult: A Case Report. JBJS Case Connect 9 (4):e0026. doi:10.2106/JBJS.CC.19.00026

41. Papachristos IV, Johnson JP, Giannoudis PV (2019) Treatment of Incarcerated Impaction of Acetabular Fractures With Concomitant Osteochondral Femoral Head Fractures by the Use of a Posterior Wall Osteotomy and Surgical Hip Dislocation: A Novel Technique. J Am Acad Orthop Surg 27 (24):e1086-e1092. doi:10.5435/JAAOS-D-18-00789

42. Masquijo JJ, Allende V, D'Elia M, Miranda G, Fernandez CA (2019) Treatment of Slipped Capital Femoral Epiphysis With the Modified Dunn Procedure: A Multicenter Study. J Pediatr Orthop 39 (2):71-75. doi:10.1097/BPO.0000000000000936

43. Liu Q, He HB, Zeng H, Yuan YH, Long F, Tian J, Luo W (2019) Modified trapdoor procedures by surgical dislocation approach to treat chondroblastoma of the femoral head. Bone Joint J 101-B (6):732-738. doi:10.1302/0301-620X.101B6.BJJ-2018-1599.R1

44. Leunig M, Fickert S, Ganz R (2019) Findings and Management of the Rare Caput Flexum Deformity of the Hip: A Case Report. JBJS Case Connect 9 (3):e0321. doi:10.2106/JBJS.CC.18.00321

45. Kim HT, Kim UJ, Cho YJ (2019) Anterolateral Approach in the Treatment of Femoroacetabular Impingement of the Hip. Clin Orthop Surg 11 (3):337-343. doi:10.4055/cios.2019.11.3.337

46. Haefeli PC, Tannast M, Beck M, Siebenrock KA, Buchler L (2019) Subchondral drilling for chondral flaps reduces the risk of total hip arthroplasty in femoroacetabular impingement surgery at minimum five years follow-up. Hip Int 29 (2):191-197. doi:10.1177/1120700018781807

47. Ahmad SS, Heilgemeir M, Anwander H, Beck M (2019) Surgical hip dislocation is more powerful than arthroscopy for achieving high degrees of acetabular correction in pincer type impingement. Orthop Traumatol Surg Res 105 (7):1339-1344. doi:10.1016/j.otsr.2019.08.009

48. Trikha V, Das S, Madegowda A, Agrawal P (2018) Midterm results of trochanteric flip osteotomy technique for management of fractures around the hip. Hip Int 28 (2):148-155. doi:10.5301/hipint.5000539

49. Rego PA, Mascarenhas V, Oliveira FS, Pinto PC, Sampaio E, Monteiro J (2018) Arthroscopic versus open treatment of cam-type femoro-acetabular impingement: retrospective cohort clinical study. Int Orthop 42 (4):791-797. doi:10.1007/s00264-017-3735-4

50. Hufeland M, Gesslein M, Perka C, Schroder JH (2018) Long-term outcome of pigmented villonodular synovitis of the hip after joint preserving therapy. Arch Orthop Trauma Surg 138 (4):471-477. doi:10.1007/s00402-017-2864-y

51. Ahmad S, Qadir I, Zaman AU, Khan CM, Javed S, Ahmad N, Aziz A (2018) Capsular arthroplasty for neglected developmental dysplasia of hip. J Orthop Surg (Hong Kong) 26 (2):2309499018777888. doi:10.1177/2309499018777888

52. Zurmuhle CA, Anwander H, Albers CE, Hanke MS, Steppacher SD, Siebenrock KA, Tannast M (2017) Periacetabular Osteotomy Provides Higher Survivorship Than Rim Trimming for Acetabular Retroversion. Clin Orthop Relat Res 475 (4):1138-1150. doi:10.1007/s11999-016-5177-5

53. Yao C, Yi N, Shen J, Du B, Sun G, Shu H, Zhang C (2017) Clinical reports of surgical dislocation of the hip with sequestrum clearance and impacting bone graft for grade IIIA-IIIB aseptic necrosis of femoral head (ANFH) patients. Oncotarget 8 (30):50084-50090. doi:10.18632/oncotarget.15095

54. Vaishya R, Vijay V, Swami S, Vaish A (2017) Isolated acetabular osteochondroma of the hip. Acta Orthop Traumatol Turc 51 (5):420-424. doi:10.1016/j.aott.2015.12.001

55. Schweitzer D, Klaber I, Zamora T, Amenabar PP, Botello E (2017) Surgical dislocation of the hip without trochanteric osteotomy. J Orthop Surg (Hong Kong) 25 (1):2309499016684414. doi:10.1177/2309499016684414

56. Haefeli PC, Marecek GS, Keel MJ, Siebenrock KA, Tannast M (2017) Patients undergoing surgical hip dislocation for the treatment of acetabular fractures show favourable long-term outcome. Bone Joint J 99-B (4):508-515. doi:10.1302/0301-620X.99B4.37681

57. Haefeli PC, Albers CE, Steppacher SD, Tannast M, Buchler L (2017) What Are the Risk Factors for Revision Surgery After Hip Arthroscopy for Femoroacetabular Impingement at 7-year Followup? Clin Orthop Relat Res 475 (4):1169-1177. doi:10.1007/s11999-016-5115-6

58. Guindani N, Eberhardt O, Wirth T, Surace MF, Fernandez FF (2017) Surgical dislocation for pediatric and adolescent hip deformity: clinical and radiographical results at 3 years follow-up. Arch Orthop Trauma Surg 137 (4):471-479. doi:10.1007/s00402-017-2644-8

59. Anwander H, Siebenrock KA, Tannast M, Steppacher SD (2017) Labral Reattachment in Femoroacetabular Impingement Surgery Results in Increased 10-year Survivorship Compared With Resection. Clin Orthop Relat Res 475 (4):1178-1188. doi:10.1007/s11999-016-5114-7

60. Won Y, Lee GS, Kim SB, Kim SJ, Yang KH (2016) Osteochondral Autograft from the Ipsilateral Femoral Head by Surgical Dislocation for Treatment of Femoral Head Fracture Dislocation: A Case Report. Yonsei Med J 57 (6):1527-1530. doi:10.3349/ymj.2016.57.6.1527

61. Van Nortwick S, Beck N, Li M (2016) Adolescent Hip Fracture-Dislocation: Transphyseal Fracture with Posterior Dislocation of the Proximal Femoral Epiphysis: A Case Report. JBJS Case Connect 6 (3):e62. doi:10.2106/JBJS.CC.15.00271

62. Sorel JC, Facee Schaeffer M, Homan AS, Scholtes VA, Kempen DH, Ham SJ (2016) Surgical hip dislocation according to Ganz for excision of osteochondromas in patients with multiple hereditary exostoses. Bone Joint J 98-B (2):260-265. doi:10.1302/0301-620X.98B2.36521

63. Snoap T, Freyder J, Roberts J (2016) Management of a Combined Femoral Head and Neck Fracture: A Case Report. JBJS Case Connect 6 (4):e88. doi:10.2106/JBJS.CC.16.00066

64. Rudiger HA, Piasecki K, Becce F, Cherix S (2016) Trans-foveal approach for curettage and bone grafting of a giant cell tumour in the femoral head. Hip Int 26 (6):612-614. doi:10.5301/hipint.5000398

65. Probe R (2016) Surgical Dislocation of the Hip in the Treatment of Complex Osteochondral Injury to the Femoral Head. J Orthop Trauma 30 Suppl 2:S17-18. doi:10.1097/BOT.0000000000000608

66. Novais EN, Heare TC, Hill MK, Mayer SW (2016) Surgical Hip Dislocation for the Treatment of Intra-Articular Injuries and Hip Instability Following Traumatic Posterior Dislocation in Children and Adolescents. J Pediatr Orthop 36 (7):673-679. doi:10.1097/BPO.0000000000000527

67. Maqungo S, Hoppe S, Kauta JN, McCollum GA, Laubscher M, Held M, Keel MJB (2016) Surgical hip dislocation for removal of retained intra-articular bullets. Injury 47 (10):2218-2222. doi:10.1016/j.injury.2016.06.020

68. Louahem D, Lozach F, Delpont M, Weiss A, Prodhomme O, Cottalorda J (2016) Mosaicplasty for femoral osteochondritis dissecans. Orthop Traumatol Surg Res 102 (2):247-250. doi:10.1016/j.otsr.2015.12.013

69. Inan U, Harmansa S, Omeroglu H (2016) Treatment of mixed type femoroacetabular impingement using safe surgical hip dislocation in adults. Eklem Hastalik Cerrahisi 27 (3):160-166. doi:10.5606/ehc.2016.32

70. Hanke MS, Steppacher SD, Zurmuhle CA, Siebenrock KA, Tannast M (2016) Hips With Protrusio Acetabuli Are at Increased Risk for Failure After Femoroacetabular Impingement Surgery: A 10-year Followup. Clin Orthop Relat Res 474 (10):2168-2180. doi:10.1007/s11999-016-4918-9

71. Eid MA (2016) Hip preservation surgery for adolescents and young adults with Post-Perthes Sequelae. Acta Orthop Belg 82 (4):821-828

72. Balakumar B, Madan S (2016) Late correction of neck deformity in healed severe slipped capital femoral epiphysis: short-term clinical outcomes. Hip Int 26 (4):344-349. doi:10.5301/hipint.5000347

73. Abdelazeem AH, Beder FK, Abdel Karim MM, Abdelazeem H, Abdel-Ghani H (2016) The anatomical reduction of a moderate or severe stable slipped capital femoral epiphysis by modified Dunn subcapital osteotomy using the Ganz approach: functional and radiological outcomes. Bone Joint J 98-B (9):1283-1288. doi:10.1302/0301-620X.98B9.37071

74. Steppacher SD, Anwander H, Zurmuhle CA, Tannast M, Siebenrock KA (2015) Eighty percent of patients with surgical hip dislocation for femoroacetabular impingement have a good clinical result without osteoarthritis progression at 10 years. Clin Orthop Relat Res 473 (4):1333-1341. doi:10.1007/s11999-014-4025-8

75. Podeszwa DA, De La Rocha A, Larson AN, Sucato DJ (2015) Surgical Hip Dislocation is Safe and Effective Following Acute Traumatic Hip Instability in the Adolescent. J Pediatr Orthop 35 (5):435-442. doi:10.1097/BPO.0000000000000316

76. Mladenovic M, Andjelkovic Z, Micic I, Mladenovic D, Stojiljkovic P, Milenkovic T (2015) Surgical dislocation of the hip in patients with femoroacetabular impingement: Surgical techniques and our experience. Vojnosanit Pregl 72 (11):1004-1009. doi:10.2298/vsp131207097m

77. Masse A, Aprato A, Alluto C, Favuto M, Ganz R (2015) Surgical hip dislocation is a reliable approach for treatment of femoral head fractures. Clin Orthop Relat Res 473 (12):3744-3751. doi:10.1007/s11999-015-4352-4

78. Hingsammer AM, Lee CB, LaReau J, Kalish LA, Kim YJ (2015) Is acetabular osteoplasty always required in mixed impingement? Eur J Orthop Surg Traumatol 25 (2):331-338. doi:10.1007/s00590-014-1507-z

79. Gavaskar AS, Tummala NC (2015) Ganz Surgical Dislocation of the Hip Is a Safe Technique for Operative Treatment of Pipkin Fractures. Results of a Prospective Trial. J Orthop Trauma 29 (12):544-548. doi:10.1097/BOT.0000000000000399

80. Gagala J, Tarczynska M, Gaweda K (2015) Mosaicplasty for the treatment of osteochondritis dissecans following Legg-Calve-Perthes disease: a case report and literature review. Acta Orthop Traumatol Turc 49 (6):694-697. doi:10.3944/AOTT.2015.13.0012

81. Clohisy JC, Nepple JJ, Ross JR, Pashos G, Schoenecker PL (2015) Does surgical hip dislocation and periacetabular osteotomy improve pain in patients with Perthes-like deformities and acetabular dysplasia? Clin Orthop Relat Res 473 (4):1370-1377. doi:10.1007/s11999-014-4115-7

82. Bilge O, Doral MN, Yel M, Karalezli N, Miniaci A (2015) Treatment of osteonecrosis of the femoral head with focal anatomic-resurfacing implantation (HemiCAP): preliminary results of an alternative option. J Orthop Surg Res 10:56. doi:10.1186/s13018-015-0199-3

83. Beaule PE, Singh A, Poitras S, Parker G (2015) Surgical Dislocation of the Hip for the Treatment of Pre-Arthritic Hip Disease. J Arthroplasty 30 (9):1502-1505. doi:10.1016/j.arth.2015.04.009

84. Steppacher SD, Huemmer C, Schwab JM, Tannast M, Siebenrock KA (2014) Surgical hip dislocation for treatment of femoroacetabular impingement: factors predicting 5-year survivorship. Clin Orthop Relat Res 472 (1):337-348. doi:10.1007/s11999-013-3268-0

85. Novais EN, Heyworth BE, Stamoulis C, Sullivan K, Millis MB, Kim YJ (2014) Open surgical treatment of femoroacetabular impingement in adolescent athletes: preliminary report on improvement of physical activity level. J Pediatr Orthop 34 (3):287-294. doi:10.1097/BPO.0000000000000093

86. Mladenovic D, Andjelkovic Z, Vukasinovic Z, Mitkovic M, Milenkovic S, Micic I, Mladenovic M (2014) Early clinical results of surgical treatment of patients with femoroacetabular impingement. Srp Arh Celok Lek 142 (5-6):325-329. doi:10.2298/sarh1406325m

87. Meulenkamp B, Gravel D, Beaule PE (2014) Viability assessment of the chondral flap in patients with cam-type femoroacetabular impingement: a preliminary report. Can J Surg 57 (1):44-48. doi:10.1503/cjs.003513

88. McLawhorn AS, Bansal M, Swensen S, Schneider R, Kelly BT, Williams RJ, 3rd, Sink EL (2014) Fibromyxoid pseudotumor of the ligamentum teres treated with fresh osteochondral allograft. Skeletal Radiol 43 (4):541-546. doi:10.1007/s00256-013-1752-y

89. Maini L, Batra S, Arora S, Singh S, Kumar S, Gautam VK (2014) Surgical dislocation of the hip for reduction of acetabular fracture and evaluation of chondral damage. J Orthop Surg (Hong Kong) 22 (1):18-23. doi:10.1177/230949901402200107

90. Khanna V, Tushinski DM, Drexler M, Backstein DB, Gross AE, Safir OA, Kuzyk PR (2014) Cartilage restoration of the hip using fresh osteochondral allograft: resurfacing the potholes. Bone Joint J 96-B (11 Supple A):11-16. doi:10.1302/0301-620X.96B11.34734

91. Gagala J, Tarczynska M, Gaweda K (2014) Fixation of femoral head fractures with autologous osteochondral transfer (mosaicplasty). J Orthop Trauma 28 (9):e226-230. doi:10.1097/BOT.0000000000000079

92. Botser IB, Jackson TJ, Smith TW, Leonard JP, Stake CE, Domb BG (2014) Open surgical dislocation versus arthroscopic treatment of femoroacetabular impingement. Am J Orthop (Belle Mead NJ) 43 (5):209-214

93. Bali K, Railton P, Kiefer GN, Powell JN (2014) Subcapital osteotomy of the femoral neck for patients with healed slipped capital femoral epiphysis. Bone Joint J 96-B (11):1441-1448. doi:10.1302/0301-620X.96B11.33519

94. Abolghasemian M, Gharanizadeh K, Kuzyk P, Masdari Z, Fakharian M, Safir O (2014) Hips with synovial chondromatosis may display the features of femoroacetabular impingement. J Bone Joint Surg Am 96 (2):e11. doi:10.2106/JBJS.L.01550

95. Zingg PO, Ulbrich EJ, Buehler TC, Kalberer F, Poutawera VR, Dora C (2013) Surgical hip dislocation versus hip arthroscopy for femoroacetabular impingement: clinical and morphological short-term results. Arch Orthop Trauma Surg 133 (1):69-79. doi:10.1007/s00402-012-1616-2

96. Sink EL, Fabricant PD, Pan Z, Dayton MR, Novais E (2013) Results of treatment of femoroacetabular impingement in adolescents with a surgical hip dislocation approach. Clin Orthop Relat Res 471 (8):2563-2569. doi:10.1007/s11999-013-3004-9

97. Masse A, Aprato A, Rollero L, Bersano A, Ganz R (2013) Surgical dislocation technique for the treatment of acetabular fractures. Clin Orthop Relat Res 471 (12):4056-4064. doi:10.1007/s11999-013-3228-8

98. Madan SS, Cooper AP, Davies AG, Fernandes JA (2013) The treatment of severe slipped capital femoral epiphysis via the Ganz surgical dislocation and anatomical reduction: a prospective study. Bone Joint J 95-B (3):424-429. doi:10.1302/0301-620X.95B3.30113

99. Kosashvili Y, Raz G, Backstein D, Lulu OB, Gross AE, Safir O (2013) Fresh-stored osteochondral allografts for the treatment of femoral head defects: surgical technique and preliminary results. Int Orthop 37 (6):1001-1006. doi:10.1007/s00264-013-1868-7

100. Gunel U, Daglar B, Gunel N (2013) Long-term follow-up of a hip joint osteoblastoma after intralesional curettage and cement packing: a case report. Acta Orthop Traumatol Turc 47 (3):218-222. doi:10.3944/aott.2013.2734

101. Domb BG, Stake CE, Botser IB, Jackson TJ (2013) Surgical dislocation of the hip versus arthroscopic treatment of femoroacetabular impingement: a prospective matched-pair study with average 2-year follow-up. Arthroscopy 29 (9):1506-1513. doi:10.1016/j.arthro.2013.06.010

102. Costa Rocha P, Klingenstein G, Ganz R, Kelly BT, Leunig M (2013) Circumferential reconstruction of severe acetabular labral damage using hamstring allograft: surgical technique and case series. Hip Int 23 Suppl 9:S42-53. doi:10.5301/HIP.2013.11662

103. Buchler L, Neumann M, Schwab JM, Iselin L, Tannast M, Beck M (2013) Arthroscopic versus open cam resection in the treatment of femoroacetabular impingement. Arthroscopy 29 (4):653-660. doi:10.1016/j.arthro.2012.12.009

104. Anderson LA, Gililland JM, Pelt CE, Peters CL (2013) Subcapital correction osteotomy for malunited slipped capital femoral epiphysis. J Pediatr Orthop 33 (4):345-352. doi:10.1097/BPO.0b013e31827d7e06

105. Walker JA, Pagnotto M, Trousdale RT, Sierra RJ (2012) Preliminary pain and function after labral reconstruction during femoroacetabular impingement surgery. Clin Orthop Relat Res 470 (12):3414-3420. doi:10.1007/s11999-012-2506-1

106. Shore BJ, Novais EN, Millis MB, Kim YJ (2012) Low early failure rates using a surgical dislocation approach in healed Legg-Calve-Perthes disease. Clin Orthop Relat Res 470 (9):2441-2449. doi:10.1007/s11999-011-2187-1

107. Ross JR, Nepple JJ, Baca G, Schoenecker PL, Clohisy JC (2012) Intraarticular abnormalities in residual Perthes and Perthes-like hip deformities. Clin Orthop Relat Res 470 (11):2968-2977. doi:10.1007/s11999-012-2375-7

108. Naal FD, Miozzari HH, Schar M, Hesper T, Notzli HP (2012) Midterm results of surgical hip dislocation for the treatment of femoroacetabular impingement. Am J Sports Med 40 (7):1501-1510. doi:10.1177/0363546512445884

109. Masse A, Aprato A, Grappiolo G, Turchetto L, Campacci A, Ganz R (2012) Surgical hip dislocation for anatomic reorientation of slipped capital femoral epiphysis: preliminary results. Hip Int 22 (2):137-144. doi:10.5301/HIP.2012.9208

110. Manner HM, Mast NH, Ganz R, Leunig M (2012) Potential contribution of femoroacetabular impingement to recurrent traumatic hip dislocation. J Pediatr Orthop B 21 (6):574-578. doi:10.1097/BPB.0b013e328357bf04

111. Li M, Luettringhaus T, Walker KR, Cole PA (2012) Operative treatment of femoral neck osteochondroma through a digastric approach in a pediatric patient: a case report and review of the literature. J Pediatr Orthop B 21 (3):230-234. doi:10.1097/BPB.0b013e3283524bc3

112. Leunig M, Tibor LM, Naal FD, Ganz R, Steinwachs MR (2012) Surgical technique: Second-generation bone marrow stimulation via surgical dislocation to treat hip cartilage lesions. Clin Orthop Relat Res 470 (12):3421-3431. doi:10.1007/s11999-012-2466-5

113. Ganz R, Slongo T, Siebenrock KA, Turchetto L, Leunig M (2012) Surgical technique: The capsular arthroplasty: a useful but abandoned procedure for young patients with developmental dysplasia of the hip. Clin Orthop Relat Res 470 (11):2957-2967. doi:10.1007/s11999-012-2444-y

114. Emre TY, Cift H, Seyhan B, Ceyhan E, Uzun M (2012) Mosaicplasty for the treatment of the osteochondral lesion in the femoral head. Bull NYU Hosp Jt Dis 70 (4):288-290

115. Albers CE, Steppacher SD, Ganz R, Siebenrock KA, Tannast M (2012) Joint-preserving surgery improves pain, range of motion, and abductor strength after Legg-Calve-Perthes disease. Clin Orthop Relat Res 470 (9):2450-2461. doi:10.1007/s11999-012-2345-0

116. Nepple JJ, Schoenecker PL, Clohisy JC (2011) Treatment of posttraumatic labral interposition with surgical hip dislocation and labral repair. Iowa Orthop J 31:187-192

117. Naal FD, Miozzari HH, Wyss TF, Notzli HP (2011) Surgical hip dislocation for the treatment of femoroacetabular impingement in high-level athletes. Am J Sports Med 39 (3):544-550. doi:10.1177/0363546510387263

118. Leunig M, Ganz R (2011) Relative neck lengthening and intracapital osteotomy for severe Perthes and Perthes-like deformities. Bull NYU Hosp Jt Dis 69 Suppl 1:S62-67

119. Kempthorne JT, Armour PC, Rietveld JA, Hooper GJ (2011) Surgical dislocation of the hip and the management of femoroacetabular impingement: results of the Christchurch experience. ANZ J Surg 81 (6):446-450. doi:10.1111/j.1445-2197.2010.05489.x

120. Jager M, Bittersohl B, Zilkens C, Hosalkar HS, Stefanovska K, Kurth S, Krauspe R (2011) Surgical hip dislocation in symptomatic cam femoroacetabular impingement: what matters in early good results? Eur J Med Res 16 (5):217-222. doi:10.1186/2047-783x-16-5-217

121. Huber H, Dora C, Ramseier LE, Buck F, Dierauer S (2011) Adolescent slipped capital femoral epiphysis treated by a modified Dunn osteotomy with surgical hip dislocation. J Bone Joint Surg Br 93 (6):833-838. doi:10.1302/0301-620X.93B6.25849

122. Girard J, Roumazeille T, Sakr M, Migaud H (2011) Osteochondral mosaicplasty of the femoral head. Hip Int 21 (5):542-548. doi:10.5301/HIP.2011.8659

123. Botser IB, Smith TW, Jr., Nasser R, Domb BG (2011) Open surgical dislocation versus arthroscopy for femoroacetabular impingement: a comparison of clinical outcomes. Arthroscopy 27 (2):270-278. doi:10.1016/j.arthro.2010.11.008

124. Bedi A, Zaltz I, De La Torre K, Kelly BT (2011) Radiographic comparison of surgical hip dislocation and hip arthroscopy for treatment of cam deformity in femoroacetabular impingement. Am J Sports Med 39 Suppl:20S-28S. doi:10.1177/0363546511412734

125. Beck M, Buchler L (2011) Prevalence and impact of pain at the greater trochanter after open surgery for the treatment of femoro-acetabular impingement. J Bone Joint Surg Am 93 Suppl 2:66-69. doi:10.2106/JBJS.J.01718

126. Tannast M, Kruger A, Mack PW, Powell JN, Hosalkar HS, Siebenrock KA (2010) Surgical dislocation of the hip for the fixation of acetabular fractures. J Bone Joint Surg Br 92 (6):842-852. doi:10.1302/0301-620X.92B6.22994

127. Slongo T, Kakaty D, Krause F, Ziebarth K (2010) Treatment of slipped capital femoral epiphysis with a modified Dunn procedure. J Bone Joint Surg Am 92 (18):2898-2908. doi:10.2106/JBJS.I.01385

128. Sink EL, Zaltz I, Heare T, Dayton M (2010) Acetabular cartilage and labral damage observed during surgical hip dislocation for stable slipped capital femoral epiphysis. J Pediatr Orthop 30 (1):26-30. doi:10.1097/BPO.0b013e3181c6b37a

129. Peters CL, Schabel K, Anderson L, Erickson J (2010) Open treatment of femoroacetabular impingement is associated with clinical improvement and low complication rate at short-term followup. Clin Orthop Relat Res 468 (2):504-510. doi:10.1007/s11999-009-1152-8

130. Naranje S, Shamshery P, Yadav CS, Gupta V, Nag HL (2010) Digastric trochanteric flip osteotomy and surgical dislocation of hip in the management of acetabular fractures. Arch Orthop Trauma Surg 130 (1):93-101. doi:10.1007/s00402-009-0873-1

131. Keel MJ, Bastian JD, Buchler L, Siebenrock KA (2010) Surgical dislocation of the hip for a locked traumatic posterior dislocation with associated femoral neck and acetabular fractures. J Bone Joint Surg Br 92 (3):442-446. doi:10.1302/0301-620X.92B3.23016

132. Bastian JD, Buchler L, Meyer DC, Siebenrock KA, Keel MJ (2010) Surgical hip dislocation for osteochondral transplantation as a salvage procedure for a femoral head impaction fracture. J Orthop Trauma 24 (12):e113-118. doi:10.1097/BOT.0b013e3181dfbb52

133. Anderson LA, Erickson JA, Severson EP, Peters CL (2010) Sequelae of Perthes disease: treatment with surgical hip dislocation and relative femoral neck lengthening. J Pediatr Orthop 30 (8):758-766. doi:10.1097/BPO.0b013e3181fcbaaf

134. Yun HH, Shon WY, Yun JY (2009) Treatment of femoroacetabular impingement with surgical dislocation. Clin Orthop Surg 1 (3):146-154. doi:10.4055/cios.2009.1.3.146

135. Van Stralen RA, Haverkamp D, Van Bergen CJ, Eijer H (2009) Partial resurfacing with varus osteotomy for an osteochondral defect of the femoral head. Hip Int 19 (1):67-70. doi:10.1177/112070000901900113

136. Solberg BD, Moon CN, Franco DP (2009) Use of a trochanteric flip osteotomy improves outcomes in Pipkin IV fractures. Clin Orthop Relat Res 467 (4):929-933. doi:10.1007/s11999-008-0505-z

137. Sierra RJ, Trousdale RT (2009) Labral reconstruction using the ligamentum teres capitis: report of a new technique. Clin Orthop Relat Res 467 (3):753-759. doi:10.1007/s11999-008-0633-5

138. Shin SJ, Kwak HS, Cho TJ, Park MS, Yoo WJ, Chung CY, Choi IH (2009) Application of Ganz surgical hip dislocation approach in pediatric hip diseases. Clin Orthop Surg 1 (3):132-137. doi:10.4055/cios.2009.1.3.132

139. Shin SJ, Choi IH, Cho TJ, Yoo WJ, Chung CY, Park MS (2009) Unusual osteocartilaginous prominence or bump causing femoroacetabular impingement after septic arthritis of the hip: a report of 2 cases in preadolescence. J Pediatr Orthop 29 (5):459-462. doi:10.1097/BPO.0b013e3181aa2465

140. Rebello G, Spencer S, Millis MB, Kim YJ (2009) Surgical dislocation in the management of pediatric and adolescent hip deformity. Clin Orthop Relat Res 467 (3):724-731. doi:10.1007/s11999-008-0591-y

141. Padhy D, Park SW, Jeong WK, Lee DH, Park JH, Han SB (2009) Femoroacetabular impingement due to synovial chondromatosis of the hip joint. Orthopedics 32 (12):921. doi:10.3928/01477447-20091020-30

142. Nakamura Y, Echigoya N, Toh S (2009) Synovial osteochondromatosis of the hip treated through a surgical dislocation. J Arthroplasty 24 (7):1143 e1115-1149. doi:10.1016/j.arth.2008.09.004

143. Khan RM, Cooper G, Hull JB (2009) Hip resurfacing through a modified anterolateral approach, as compared with the Ganz trochanteric flip osteotomy: a two year follow-up study. Hip Int 19 (4):338-342. doi:10.1177/112070000901900407

144. Jellicoe P, Son-Hing J, Hopyan S, Thompson GH (2009) Surgical hip dislocation for removal of intraarticular exostoses: report of two cases. J Pediatr Orthop 29 (4):327-330. doi:10.1097/BPO.0b013e3181a56b4f

145. Graves ML, Mast JW (2009) Femoroacetabular impingement: do outcomes reliably improve with surgical dislocations? Clin Orthop Relat Res 467 (3):717-723. doi:10.1007/s11999-008-0648-y

146. Beaule PE, Shim P, Banga K (2009) Clinical experience of Ganz surgical dislocation approach for metal-on-metal hip resurfacing. J Arthroplasty 24 (6 Suppl):127-131. doi:10.1016/j.arth.2009.04.006

147. Anderson LA, Crofoot CD, Erickson JA, Peters CL (2009) Staged surgical dislocation and redirectional periacetabular osteotomy: a report of five cases. J Bone Joint Surg Am 91 (10):2469-2476. doi:10.2106/JBJS.H.00066

148. Leunig M, Slongo T, Kleinschmidt M, Ganz R (2007) Subcapital correction osteotomy in slipped capital femoral epiphysis by means of surgical hip dislocation. Oper Orthop Traumatol 19 (4):389-410. doi:10.1007/s00064-007-1213-7

149. Espinosa N, Beck M, Rothenfluh DA, Ganz R, Leunig M (2007) Treatment of femoro-acetabular impingement: preliminary results of labral refixation. Surgical technique. J Bone Joint Surg Am 89 Suppl 2 Pt.1:36-53. doi:10.2106/JBJS.F.01123

150. Beaule PE, Le Duff MJ, Zaragoza E (2007) Quality of life following femoral head-neck osteochondroplasty for femoroacetabular impingement. J Bone Joint Surg Am 89 (4):773-779. doi:10.2106/JBJS.F.00681

151. Spencer S, Millis MB, Kim YJ (2006) Early results of treatment of hip impingement syndrome in slipped capital femoral epiphysis and pistol grip deformity of the femoral head-neck junction using the surgical dislocation technique. J Pediatr Orthop 26 (3):281-285. doi:10.1097/01.bpo.0000217726.16417.74

152. Schoeniger R, Naudie DD, Siebenrock KA, Trousdale RT, Ganz R (2006) Modified complete synovectomy prevents recurrence in synovial chondromatosis of the hip. Clin Orthop Relat Res 451:195-200. doi:10.1097/01.blo.0000229280.53109.d5

153. Peters CL, Erickson JA (2006) Treatment of femoro-acetabular impingement with surgical dislocation and debridement in young adults. J Bone Joint Surg Am 88 (8):1735-1741. doi:10.2106/JBJS.E.00514

154. Jager M, Begg MJ, Krauspe R (2006) Partial hemi-resurfacing of the hip joint--a new approach to treat local osteochondral defects? Biomed Tech (Berl) 51 (5-6):371-376. doi:10.1515/BMT.2006.072

155. Espinosa N, Rothenfluh DA, Beck M, Ganz R, Leunig M (2006) Treatment of femoro-acetabular impingement: preliminary results of labral refixation. J Bone Joint Surg Am 88 (5):925-935. doi:10.2106/JBJS.E.00290

156. Gardner MJ, Suk M, Pearle A, Buly RL, Helfet DL, Lorich DG (2005) Surgical dislocation of the hip for fractures of the femoral head. J Orthop Trauma 19 (5):334-342

157. Haverkamp D, Luitse JS, Eijer H (2004) Acetabular reduction osteotomy using surgical dislocation of the hip joint for treatment of a malunited acetabular fracture. Arch Orthop Trauma Surg 124 (8):527-530. doi:10.1007/s00402-004-0731-0

158. Leunig M, Sledge JB, Gill TJ, Ganz R (2003) Traumatic labral avulsion from the stable rim: a constant pathology in displaced transverse acetabular fractures. Arch Orthop Trauma Surg 123 (8):392-395. doi:10.1007/s00402-003-0575-z

159. Siebenrock KA, Gautier E, Woo AK, Ganz R (2002) Surgical dislocation of the femoral head for joint debridement and accurate reduction of fractures of the acetabulum. J Orthop Trauma 16 (8):543-552. doi:10.1097/00005131-200209000-00002

160. Ganz R, Gill TJ, Gautier E, Ganz K, Krugel N, Berlemann U (2001) Surgical dislocation of the adult hip a technique with full access to the femoral head and acetabulum without the risk of avascular necrosis. J Bone Joint Surg Br 83 (8):1119-1124. doi:10.1302/0301-620x.83b8.11964
